# Induced cell phenotype activity recording of DNA-tagged ligands†

**DOI:** 10.1039/d4cb00137k

**Published:** 2024-12-17

**Authors:** Philipp N. Sander, Jared T. Gillen Miller, Luke L. Lairson

**Affiliations:** a Department of Chemistry, The Scripps Research Institute 10550 North Torrey Pines Road La Jolla CA 92037 USA llairson@scripps.edu

## Abstract

Based on their ability to canvas vast genetic or chemical space at low cost and high speed, DNA-encoded libraries (DEL) have served to enable both genomic and small molecule discovery. Current DEL chemical library screening approaches focus primarily on *in vitro* target-based affinity or activity. Here we describe an approach to record the phenotype-based activity of DNA-encoded small molecules on their cognate barcode in living cells. We transfected chloroalkane-derivatized DNA barcodes carrying photoreleasable small molecules into cells. Following photorelease, bioactive compounds induced expression of a reporter gene cassette containing self-labeling HaloTag protein that becomes covalently modified by encoding barcodes. We demonstrate that we can recover activity information from cells that received active compound following immunoprecipitation-based enrichment. This generalizable approach should enable future strategies that facilitate phenotype-based screens of DNA-encoded chemical libraries in complex cellular or organism level systems.

## Abstract 2

A generalizable methodology for recording and retrieving the phenotype-inducing activity of DNA-encoded ligands.

## Graphical abstract

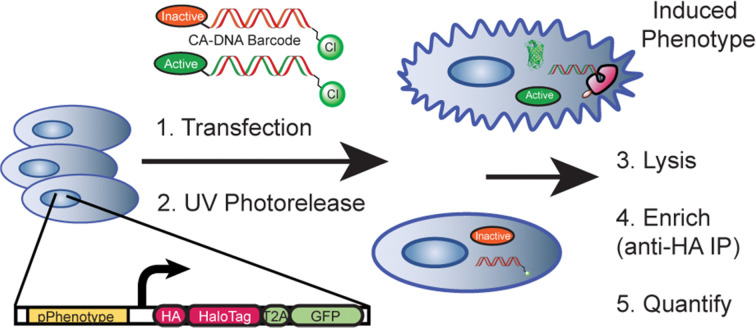

DNA-encoded perturbation assays have significantly enabled increased experimental throughput.^1–3^ Genetic screens using pooled libraries of ∼10^5^ individual barcoded shRNA or gRNA constructs have become a standard biological discovery tool,^4^ while DNA-encoded chemical libraries have transferred the pooled perturbation paradigm to the world of small molecule hit identification.^5^ Typical DNA-encoded libraries (DELs) consist of individual small molecules, generally prepared using highly scalable split and pool combinatorial synthesis,^6^ which are physically attached to a DNA barcode whose base sequence specifically encodes the chemical structure of the attached compound.^6,7^ PCR amplification and subsequent deep sequencing identifies the composition and relative abundance of a mixture (“pool”) of DNA-encoded molecules. Biochemical methods, such as affinity enrichment to partition a mixed pool of DNA-tagged small molecules into defined fractions (*e.g.* high affinity, low affinity, or non-binder with respect to a defined target or anti-target), allow for large libraries to be assayed in parallel in the same reaction volume. First proposed and described by Lerner & Brenner^8^ and subsequently reduced to practice by the Janda,^9^ Gallop,^10^ Liu,^7^ Harbury,^11^ Neri^12^ and Morgan^13^ research groups, amongst others, the natural alliance of combinatorial chemistry with combinatorial DNA encoding allowed construction and interrogation of small molecule libraries with a theoretical library size of over 1 trillion individual members.^14^ Subsequent industrialization of the process has led to broad successful adoption of DNA encoded chemical library technology within the pharmaceutical industry.^5,15^

While genetic screens are performed at the cell-based, phenotypic level, solution-phase DNA encoded chemical libraries have so far been primarily interrogated against defined targets *via* physical binding assays in buffer conditions. In the canonical workflow, an immobilized target protein is incubated with the DNA encoded library in an *in vitro* environment, whereby modest affinity small molecules bind to the target. Low or no affinity conjugates are then washed away, and after repeated washes, retained molecules are identified by DNA sequencing *via* their specific barcode.^13^ Efforts in the field have been expanding the assay scope for DNA encoded libraries to probe both binding and function in both *in vitro* and *in cellulo* settings.^16^ Affinity-based DEL selections have been successfully conducted in cell lysates,^17^ and even *Xenopus laevis* oocytes.^18^ The Krusemark group pioneered activity-based DEL selections using enzymatic activity to transfer an affinity handle onto the DNA barcode for enrichment, enabling binding based selections in living mammalian cells.^16^ Using one-bead-one-compound libraries (OBOC) with DNA tags for decoding, the Paegel lab has established an extensive body of work demonstrating both binding- and enzymatic activity-dependent DEL screening in microfluidic sorting devices,^19^ as well as the first phenotypic screen of DELs in bacterial cell killing assays on bacterial lawns.^20^ More recently, the Krusemark group achieved readout of biased GPCR activation in cells, using a split protein complementation-based approach that has broad potential applicability in the selection of chemical inducers of dimerization.^21^ However, DEL selection methods based on target agnostic phenotypic cellular activity, involving multiple cellular components (*e.g.*, induced transcriptional activity), have yet to be reported. Here we describe a generalizable approach, involving covalent capture of a cognate DNA barcode *via* induced expression of a self-labeling reporter protein, for recording and retrieving the activity of DNA-encoded small molecules in living cells based on induced cellular phenotype.

When used appropriately, reporter gene assays can serve as effective tools for phenotype-based screening and drug discovery. By linking the transcription of a reporter gene (*e.g.*, luciferase, GFP, *etc.*) to activation of the relevant transcriptional regulatory element of a gene that serves to define a given cell fate or state (*e.g.*, a key transcription factor or marker of activation state), small molecules that induce a phenotype of interest can be identified in high throughput target-agnostic fashion. Reporter gene assays have been used to successfully identify small molecule activity in diverse pathway-targeted and globally agnostic settings,^22–25^ and therefore represent a powerful and well established approach for the discovery of new chemical matter and their associated targets and mechanisms.^22,24,26,27^ Here, we sought to develop a generalizable reporter gene-based approach to facilitate the interrogation of DNA-encoded chemical libraries in living cells, based on induced phenotypes. We reasoned that coupling expression of a reporter gene, which consists of an enrichable HaloTag construct, to the cell-based activity of a small molecule that is encoded by a chloroalkane-derivatized DNA barcode (Fig. 1), we could link induced phenotype to DNA encoded chemotype.

**Fig. 1:**
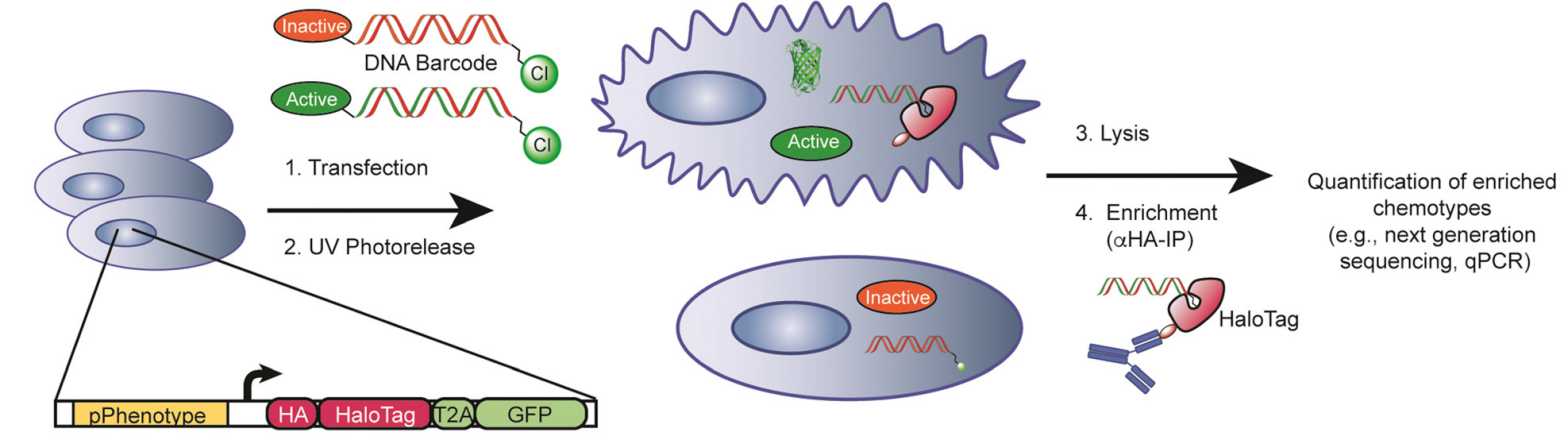
Description of the PAR-DEL workflow. Small molecules are conjugated to DNA barcodes encoding chemical identity and transfected into mammalian cells expressing a HaloTag reporter gene under control of the desired phenotype promoter (pPhenotype). DNA barcodes are transfected into cells and attached compounds are UV photoreleased from the DNA barcodes. If compounds are successful in inducing the desired phenotype, the HaloTag reporter will covalently react with the DNA barcodes. After cell lysis, DNA barcodes that delivered active compounds can be retrieved and enriched by immunoprecipitating the covalent HaloTag-DNA barcode complex. qPCR or next-generation DNA sequencing of retrieved DNA barcodes yields identity and relative cell-based activity of DNA-encoded small molecules.

We designed and generated a dual-readout reporter cassette containing an HA-tagged HaloTag^28^ protein and a separate GFP protein for parallel quantitative measurement of activity (Fig. 1 and 2A). By attaching a HaloTag-reactive chloroalkane (CA) handle to compound-studded DNA, a change in cellular phenotype induced by delivery of active small molecules will produce covalent linkages between expressed HaloTag proteins and the DNA barcodes present in an activated cell. To allow for unhindered target engagement and broad intracellular distribution, we installed a photocleavable linker moiety between the small molecule and DNA barcode at the 5′ end of the forward strand. To allow for HaloTag-mediated capture and immunoprecipitation, we installed a chloroalkane HaloTag handle on the 5′ end of the complementary reverse strand. As shown in Fig. 1, we envisioned a methodology consisting of (i) transfection of CA-DNA barcoded small molecules into cells, (ii) photorelease, (iii) phenotype-defining promoter (pPhenotype)-dependent transcription of HA-tagged HaloTag genetic reporter gene, (iv) in cell activity recording on DNA barcodes *via* covalent reaction of HaloTag reporter protein with the CA functional group on the DNA barcode, (v) immunoprecipitation of the covalent HaloTag-DNA barcode complex, and (vi) quantitative detection and deconvolution of active chemotype (*e.g.*, next generation sequencing or qPCR for proof-of-concept). With this core architecture we present a generalizable approach for cell-based evaluation of the activity of DNA encoded small molecules based on induced cellular phenotypes. We abbreviate this approach as PAR-DEL, for induced cell Phenotype Activity Recording of DNA-encoded ligands.

**Fig. 2:**
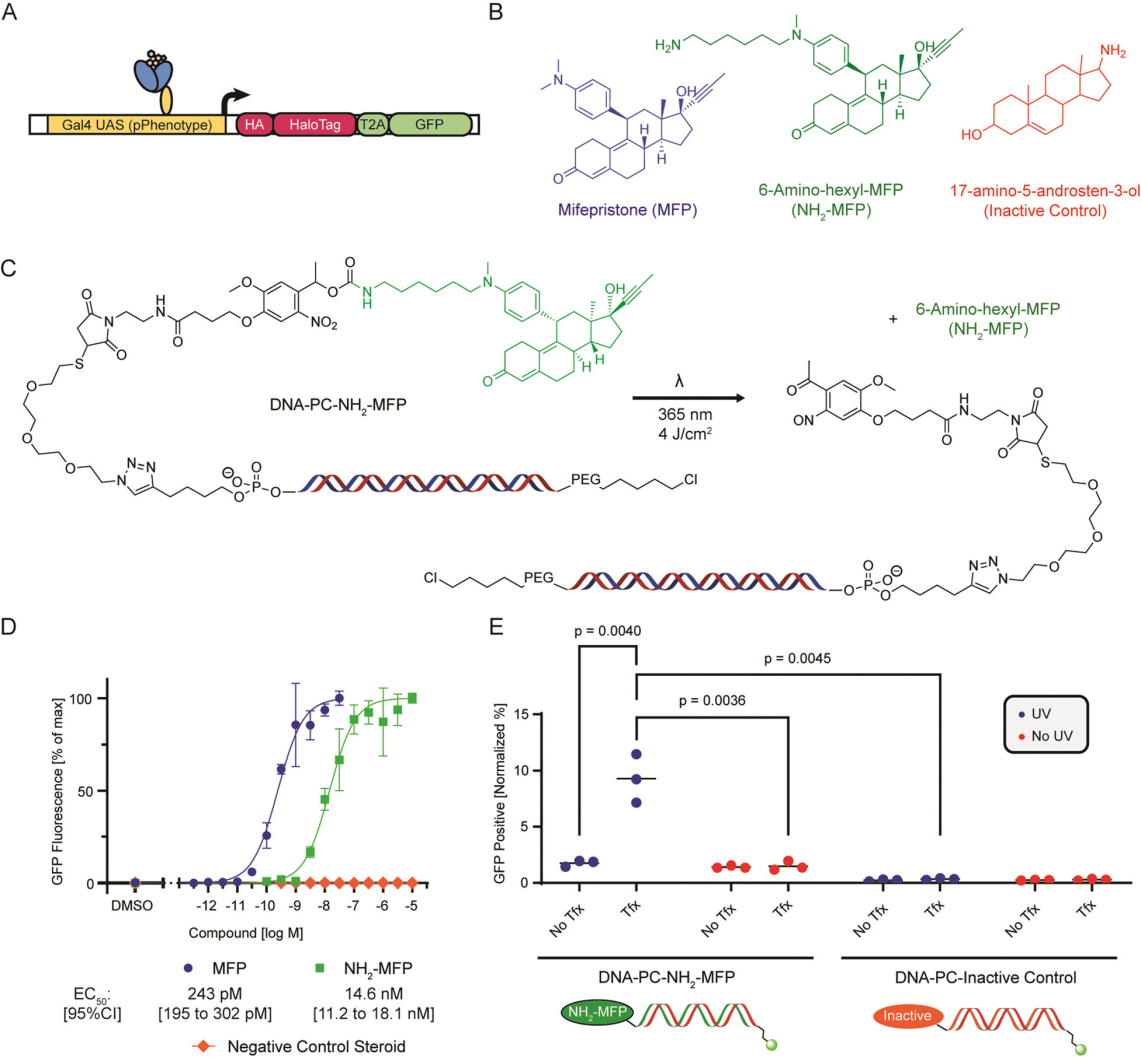
(A) Scheme of the pGene-HaloTag-GFP construct showing the MFP responsive Gal4-PR-LBD-p65 transcription factor driving expression of the 3xHA-tagged HaloTag-GFP fusion protein. (B) Structures of Mifepristone (MFP), 6-amino-hexyl-MFP (NH_2_-MFP) and 17-amino-5-adrosten-3-ol (inactive control). (C) Scheme illustrating the DNA barcode architecture and photorelease reaction. The functionalized barcode consists of a double-stranded DNA molecule (127 bp) functionalized on the reverse strand 5′ end with a chloroalkane-PEG moiety for HaloTag conjugation, and on the forward strand 5′ end a steroid compound attached through a photocleavable nitroveratyl group. Upon illumination with UV light (*λ* = 365 nm) the amino-functionalized steroid is released. (D) microscopy-based dose–response curve of small molecule-induced GFP expression of pSwitch/pGene-HaloTag-GFP transfected HEK293T cells. (E) Quantification of GFP-positive cells (previously transfected with pSwitch/pGene-HaloTag-GFP) that were treated with 10 ng DNA barcode functionalized with either NH_2_-MFP (active compound) or 17-amino-5-adrosten-3-ol (inactive control), either complexed with Lipofectamine 3000 transfection reagent (Tfx) or without (No Tfx), and exposed to 4 J cm^−2^ UV illumination or kept dark.

As a model system, we elected to utilize the GeneSwitch^TM^ (Life Technologies) reporter system that senses binding of the small molecule drug Mifepristone (MFP, Fig. 2B) to a progesterone receptor ligand binding domain (PR LBD) fused to a Gal4 activator domain.^29–31^ Upon MFP sensing, the switch protein migrates to the nucleus and induces transcription of a reporter downstream of Gal4 UAS sites, in this case our introduced HA-tagged HaloTag reporter protein (Fig. 2A). Notably, we believe that our approach will be generalizable, based on the required need to simply replace the identity of the promoter of this reporter system with one that is associated with a phenotype of interest (pPhenotype) and introducing this construct into a cell population of interest. The GeneSwitch system is relatively inert to standard cell culture conditions and shows low background signal and displays a very sensitive transcriptional response to MFP (EC_50_ = 243 pM, Fig. 2D), following transient transfection in HEK293T cells. As such, the GeneSwitch system is a well-characterized model system for small molecule-induced reporter gene induction and is an ideal system for characterizing the development of our small molecule assay method, based on the low background signal and large range of response with respect to ligand potency.

We reasoned that following transfection, barcode release might be necessary to facilitate broad intracellular distribution of ligand and sought to determine a site on the MFP sterol-based scaffold that would be tolerant to DNA barcode conjugation *via* a photocleavable linker. Based on reported SAR information associated with the development of fluorescent derivatives,^32,33^ we designed an MFP analog termed 6-amino-hexyl-MFP (NH_2_-MFP, Fig. 2B) that consists of modification at the *N*-methyl aniline position that facilitates introduction of a handle to conjugate our model small molecule payloads to DNA barcodes. While the determined cell-based activity of NH_2_-MFP displayed a moderate reduction in potency compared to the MFP parent, it retained low nM potency (EC_50_ = 14.2 nM, Fig. 2D), which we reasoned would facilitate its utility as a tool compound to characterize our general approach. A readily available primary amine-containing Androstenediol-derivative (17-amino-5-androsten-3-ol,^34^Fig. 2B) was selected as a non-active sterol-based control compound (Inactive Control, EC_50_ > 1 μM, Fig. 2D). With this cellular system and identified set of tool compounds that display concentration-dependent control of GeneSwitch transcriptional activity, we were ready to evaluate our proposed approach for enrichment-based detection of DNA-encoded chemotypes based on induced cellular phenotype.

We turned our attention to identifying optimal conditions for in-cell compound photorelease. While alternative, potentially more benign approaches to release (*e.g.*, enzyme-based) could be used in future iterations, we elected photorelease based on the readily tunable level of control it enables. The majority of reported photorelease studies have been conducted in buffer environments or in short-term cell culture experiments.^35–37^ As such, we sought to develop a methodology that would effectively release compounds in a cell culture environment while maintaining high cell viability across several days of assay duration. We selected nitroveratryl chemistry, which facilitates release following 365 nm irradiation and thus poses a reduced phototoxic impact on the cultured cells compared to other shorter-wavelength approaches. We constructed a photocleavable small-molecule-bearing DNA barcode by first reacting our amine-containing small molecules with an NHS-ester-containing nitroveratyl photocleavable linker harboring a maleimide (Mal-PC-NH_2_-MFP, Scheme S1, ESI†). To a DNA oligo containing a hex-1-yne-linker attached to the 5′ phosphate we conjugated a thiol-bearing PEG_4_ linker *via* CuAAC click chemistry. With the high reactivity of maleimide-thiol conjugations in mind, we ultimately joined the small molecule bearing photocleavable maleimide to the thiol-containing DNA in a final reaction to afford our final targets (Fig. 2C). We assembled the chloroalkane-functionalized reverse strand by reacting chloroalkane NHS-ester (Promega) with a DNA oligo bearing a terminal amino-hexyl linker attached to the 5′ phosphate. Following reverse-phase uHPLC purification, we annealed both strands to yield the highly purified final DNA barcode with specific sequences encoding the attached compound's identity (Scheme S1 and Fig. 2C, ESI†).

We next determined effective UV irradiation conditions that allow non-toxic photorelease of a conjugated small molecule in cell culture conditions. We irradiated a model photocleavable compound consisting of the hydrolyzed maleic acid derivative of Mal-PC-NH_2_-MFP in PBS with increasing amount of 365 nm UV light and determined rates of product release and substrate decay by HPLC analysis (Fig. S1, ESI†). Over 90% of compound was released upon a 4 J cm^−2^ dose of irradiation (12 min using a UVP-CL1000 instrument). To test viability of cells under these conditions, we irradiated HEK293T cells with increasing amounts of 365 nm UV light and assayed viability after 48 h. Notably, switching from phenol-red containing culture media to a basal DMEM media specially formulated for low background fluorescence (Invitrogen Fluorobrite) notably decreased photo-induced cell death. Under optimized conditions in FluoroBrite media, irradiation with 4 J cm^−2^ resulted in a minimal decrease in viability (>80% of dark-shielded cells), which was judged to be adequate for our intended experimental purposes.

Having synthesized DNA barcodes carrying either 6-amino-hexyl-MFP (active compound) or the Androstenediol-derivative (inactive control), we evaluated activity of the DNA conjugates in our GeneSwitch-based reporter assay system. We transfected HEK293T cells with plasmids for expression of both the GeneSwitch sensor (pSwitch) and the HaloTag-GFP reporter cassette (pGene-HaloTag-GFP), and subsequently transfected cells using Lipofectamine 3000 with compound-functionalized barcodes at a dose of 10 ng (2.6 nM in 50 μl well volume) of DNA barcode per well of a 96 well plate. As a control, cells were treated with barcodes without the use of transfection reagent. Following 4 hours of incubation under DNA barcode transfection conditions, cells were washed vigorously to remove DNA barcodes not delivered into the cells and then irradiated at 4 J cm^−2^ to release the compounds. As a control, a DNA-treated plate was kept in the dark and not UV irradiated. Cells were analyzed by fluorescence microscopy to quantify GFP expression following 24 hours. A significant increase in GFP expression was observed in cells treated with active compound-DNA conjugates compared to those treated with inactive compound-DNA control, which was completely dependent on the use of transfection reagent and UV irradiation (Fig. 2E). This suggested that GFP expression was driven by transfection reagent-mediated delivery of active compound to the cell interior, and that photorelease and subsequent unhindered target engagement and/or intracellular distribution was necessary to activate reporter gene expression.

We next focused on further studying and optimizing delivery of functionalized DNA into cells to identify optimal assay conditions. The field of nucleic acid transfection has focused on two general types of nucleic acid payloads – large double-stranded circular DNA (*e.g.*, plasmids) or short single-stranded linear RNA (*e.g.*, siRNA). Thus, reagents and protocols are optimized for delivery for one or both payload archetypes. To evaluate the suitability of different commercial transfection reagents in the delivery of a short, modified, linear DNA barcode, we screened potential transfection reagents (*i.e.*, Lipofectamine 3000, Polyethyleneimine (PEI), Transit-X2 and LyoVec (Fig. S2 and S3, ESI†)), representing various polymeric and lipid nanoparticle transfection technology. We transfected a rhodamine fluorophore (TMR)-labeled 127 bp DNA barcode into HEK293T cells and performed quantitative flow cytometry- or imaging-based analysis of washed cells. Based on these collective efforts (Fig. S2, ESI†), we determined that a high 30 : 1 N : P ratio of PEI facilitates rapid high intracellular DNA signal based on analysis following 4 hours of incubation, albeit at the cost of overt cytotoxicity, and that transfection with Lipofectamine 3000 for 24 hours results in a maximal level of transfection but without appreciably impacting apparent cell viability (Fig. S2B and S2C, ESI†).

We then asked if expressed HaloTag protein can react with transfected chloroalkane-functionalized DNA inside of living cells. Prior reports have studied the delivery path of transfected DNA from extracellular space through endosomal uptake, endosomal escape and subsequent entry into the cytosol and the nucleus.^38^ Most studies quantify DNA transfection success based on transcription of a DNA template delivered to the nucleus, whereas we were interested to understand if DNA is delivered to a cell compartment (*e.g.*, cytosol or nucleus) that is accessible to expressed reporter proteins. We transfected cells with an 127 bp double-stranded DNA functionalized with a Cy5 fluorophore on the 5′ end of the forward strand for detection and a chloroalkane ligand on the reverse 5′ end for HaloTag labeling (Cy5-BC-Cl, Fig. 3A). We first evaluated the localization of the Cy5/chloroalkane-functionalized DNA and observed that DNA cargo is predominantly localized to the cell body/cytosol and not to the nucleus, which agreed with localization observations for TMR-labeled DNA lacking a chloroalkane (Fig. S2A, ESI†). We first evaluated HaloTag variant HT7^28^ constructs with and without a nuclear localization tag, and characterized localization and induced expression based on imaging analysis following treatment with MFP (32 μM) and incubation with rhodamine-chloroalkane fluorophore control reagent (TMR-Cl, Fig. 3B). To assess successful reaction between HaloTag and chloroalkane-labeled DNA, following MFP treatment, we lysed cells transiently transfected with expression plasmids for the Switch protein (pSwitch) and HaloTag-GFP variants (pGene-HaloTag-GFP), and analyzed the lysate by SDS-PAGE to detect apparent molecular weight changes for the Cy5-labeled DNA. As a control for HaloTag reactivity, we separately incubated cells with TMR-Cl control substrate. Robust TMR labeling of HaloTag protein bands, based on western blot detection of the fused 3xHA tag, was observed for both HT7-based constructs. However, based on both gel shift and western blot analysis, labeling of either HT7 construct by Cy5-BC-Cl was observed to be similarly weak (HT7 or HT7-NLS, Fig. 3C). This suggested that the induced HaloTag proteins were functional in cells, but potentially that conjugation of the chloroalkane substrate to negatively charged DNA was hampering reaction efficiency. We therefore turned to a DNA-binding HaloTag mutant (HOB), which has been optimized based on observed reductions in *k*_obs_ for DNA-based substrates that presumably results from charge repulsion effects between negatively charged DNA backbones and an acidic patch surrounding the ligand entry tunnel of HaloTag protein.^39^ When compared to equivalent induced expression levels of HT7, the HOB construct was observed to be equivalently labeled by control TMR-Cl reagent, but displayed a marked increase in Cy5 staining following treatment with the Cy5-BC-Cl DNA construct (Fig. 3C). We thus decided to proceed with Lipofectamine 3000-based delivery of DNA barcodes into cells harboring a non-localized HOB reporter construct.

**Fig. 3:**
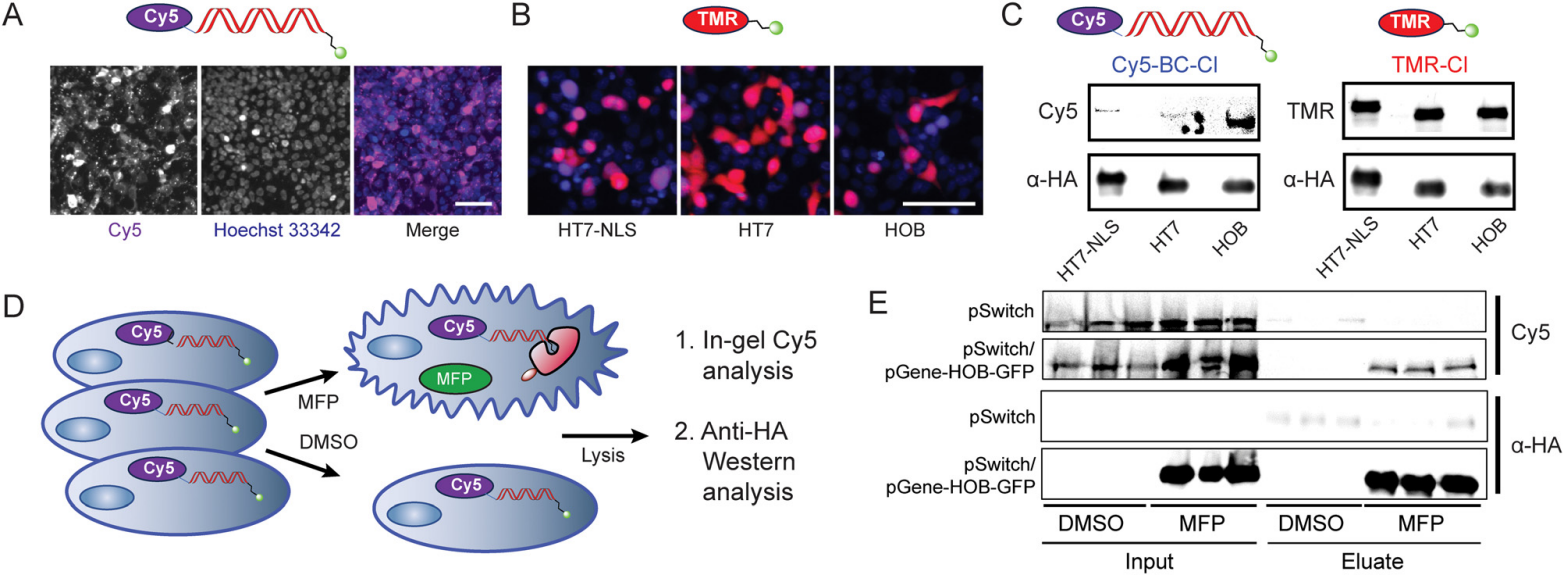
(A) Delivery of Cy5-labeled Barcode DNA to HEK293T cells imaged after 24 h at 10× magnification, right images are magnified insets. Scale bar equivalent to 100 μm. (B) Fluorescence microscopy of different HaloTag-NLS, HaloTag, and HOB constructs stained with TMR-Cl and Hoechst 33342 as nuclear counter stain. Scale bar equivalent to 100 μm. (C) Test labeling of different HaloTag constructs with chloroalkane-functionalized TMR or Cy5. Top blots show in-gel fluorescence, bottom blots show anti-HA immunoblot. (D) Scheme of transfection with fluorescently labeled DNA, in-cell conjugation to HaloTag protein (red), lysis and anti-HA immunoprecipitation, and subsequent in-gel Cy5 fluorescence detection and anti-HA western blot. (E) SDS-PAGE in-gel fluorescence and western blot analysis of fluorescently-tagged DNA from cells first transfected with pSwitch or pSwitch/pGene-HOB-GFP, then transfected with 100 ng Cy5-labeled, chloroalkane-functionalized barcode and DMSO or MFP treatment, in three biological replicates (loaded onto a single gel for direct comparison of biological variability).

Having established conditions for the delivery of modified DNA barcodes into mammalian cells, as well as for their in-cell labeling of a HaloTag protein, we turned our attention to DNA barcode retrieval. We transfected HEK293T cells with GeneSwitch sensor and HOB-GFP reporter cassette plasmids, and subsequently transfected cells with a Cy5/chloroalkane-functionalized DNA barcode (Cy5-BC-Cl) and then treated with DMSO or MFP to induce HaloTag expression (Fig. 3D). Following block of unreacted HaloTag with 7-Bromo-Heptanol and cell lysis, we performed magnetic bead-based immunoprecipitations using an anti-HA antibody to facilitate enrichment of expressed HOB HaloTag, which contains an N′-terminal 3xHA fusion tag. In the context of SDS denaturation to detect HOB protein covalently modified by the Cy5-BC-Cl DNA construct, we evaluated HOB levels in the input and eluate fractions by western blot analysis and looked for the presence of co-localized mass shifted Cy5 signal by in-gel fluorescence (Fig. 3E). Using control cells transfected with only the pSwitch GeneSwitch sensor plasmid, recovery of Cy5-labeled DNA was not detected in the eluate, independent of DMSO or MFP treatment (Fig. 3E). In contrast, in cells harboring both pSwitch and pGene-HOB-T2A-GFP plasmids, recovery of both mass-shifted Cy5-tagged DNA and HA-tagged HaloTag protein was observed in the eluate in an MFP treatment-dependent manner (Fig. 3E). Based on observed resistance to SDS denaturation, intracellular formation of a covalent linkage between compound-induced HaloTag protein and the transfected DNA barcode had occurred (Fig. 3E), which demonstrated our overall ability to recover HaloTag-coupled DNA. We had thus demonstrated that our system can recover DNA barcodes in an inducible, HaloTag reaction-dependent manner.

Finally, we evaluated quantitative, sequence-specific detection of enriched DNA barcodes in the context of our complete workflow that links recovered genotype to the cell-based activity of a chemotype. We delivered chloroalkane-containing DNA barcodes consisting of distinct base sequences functionalized with either photocleavable 6-amino-hexyl-MFP or the photocleavable inactive control compound to cells previously transfected with pSwitch/pGene-HOB-GFP (Fig. 4A). A minimally efficacious ∼EC_20_ concentration of DNA-PC-NH_2_-MFP was used to simulate reporter activation and was compared against the same concentration of DNA-PC-NH_2_-Inactive. To normalize for DNA transfection efficiency and sample loss during processing, cells uniformly received a normalization barcode as part of the same barcode transfection mix, which consisted of a unique base sequence that was functionalized with chloroalkane but lacked a photorelease compound. Following 4 hours of transfection, we released small molecule payloads by UV photoirradiation and also prepared a match-paired no-UV control plate. Following 24 hours of cell incubation, we evaluated GFP expression levels by fluorescence microscopy and observed distinct UV irradiation-dependent ∼10-fold increase in GFP expression (%GFP positive) in cells that received the active compound (NH_2_-MFP) barcode conjugate (Fig. 4B), when compared to cells treated with the inactive control compound barcode conjugate, yielding a *Z*′ factor of 0.78 (Table S3, ESI†). We immunoprecipitated HaloTag-DNA complexes from lysates of treated cells and analyzed input and eluate fractions by qPCR, using primer pairs specific for the active compound, negative control, or normalization barcode sequences. After normalizing DNA barcodes coding for active *vs.* inactive compounds to the normalization barcode, we quantified the recovery of sequence-specific barcodes from the eluate as a fraction of input sample total DNA. Using conditions that result in low level reporter activation (∼EC_20_ concentration of active barcode), we observed a clear and statistically significant increase in compound-specific sequence recovery from cells transfected with active compound conjugates and subjected to UV irradiation, when compared to either non-irradiated cells, or to cells transfected with inactive compound conjugate (Fig. 4C). Thus, we demonstrate that we can recover specific DNA sequences encoding a specific small molecule, and that this recovery correlates with the activity of the encoded molecule. This sets the stage for screening larger sets of encoded molecules in arrayed or pooled formats, which represents an advancement towards target agnostic interrogation of DNA encoded chemical space in live systems based on an induced phenotype.

**Fig. 4:**
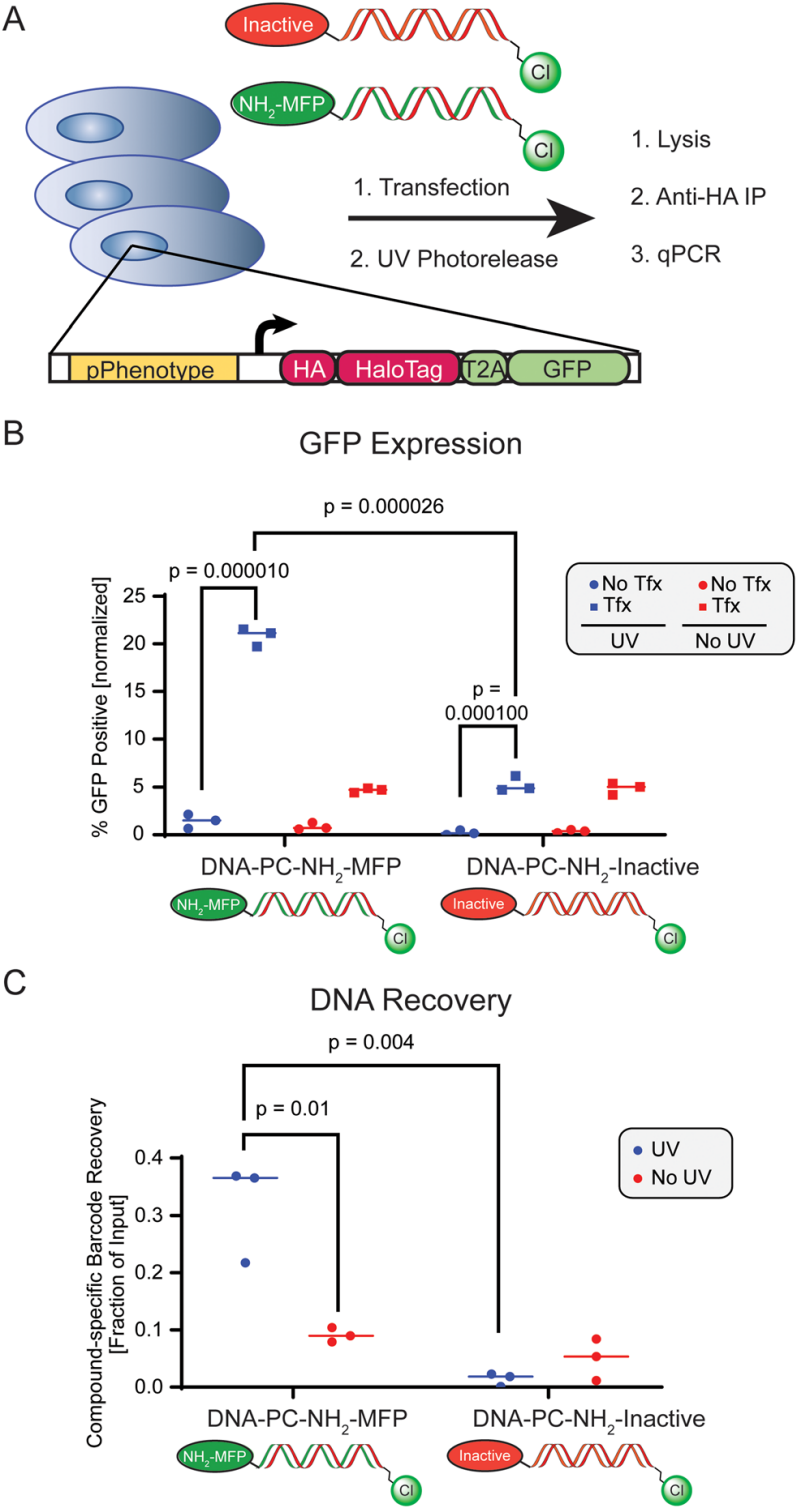
(A) Scheme of transfection of compound-loaded DNA, in-cell HaloTag reaction, immunoprecipitation from lysate and qPCR quantification. HEK293T cells previously transfected with pSwitch/pGene-HOB-GFP were transfected with 50 ng of either NH_2_-MFP (active compound)-or inactive control-functionalized DNA barcodes with compound-specific sequences. (B) GFP reporter quantification by fluorescence microscopy. (C) Recovery of compound-specific barcode as measured by qPCR for compound-functionalized and transfected barcodes.

We have demonstrated that the cellular activity of a bioactive small molecule can be recorded *via* covalent capture of a cognate DNA barcode, by employing photorelease chemistry, standard DNA lipofection, and a self-labeling protein. The current report demonstrates recovery of single DNA barcodes transfected in parallel as opposed to in a pooled format, which would be needed to enable the useful screening of a chemically diverse DEL. Physically dissociating compound-target interaction from the DNA barcode recovery mechanism poses notable challenges for the adaption to pooled DNA encoded libraries and bulk transfection-based methods (*i.e.*, the achievement of a distribution of non-equivalent but efficacious and non-toxic concentrations of unique library members in distinct cells). Potential solutions include application to DNA-encoded OBOC libraries or the use of lipid nanoparticle (LNP)-based approaches for delivery (*e.g.*, delivery of a bolus of homogeneous DNA-barcode conjugates to individual cells to allow screening of “packets” of unique DNA encoded molecules), and possibly relying on microfluidic-based delivery. Relatedly, our PAR-DEL methodology could enable experimentation in more physiologically relevant settings, including potentially screens in whole organisms, analogous to *in vivo* CRISPR screens.^40^

The utility of recording cellular events on DNA has been demonstrated in bacterial and mammalian systems.^41,42^ If we view the covalent labeling of DNA with HaloTag protein as another embodiment of activity recording, we can postulate that this might be only one of several ways to record DNA-encoded compound activity by way of physical modification of DNA barcodes. For example, it should be possible to record multiple orthogonal cellular signals by expressing a variety of DNA modifying enzymes such as TdT^43^ or other classes of self-labeling proteins (such as SNAPtags and CLIPtags)^44,45^ that enable orthogonal immunoprecipitation. Modifications that can be read out by DNA sequencing include methylation marks that can be edited by DNA methyltransferases and methyltransferases, and changes to the primary base sequence of the barcode that can be effected by DNA base editors such as AID and newly engineered variants.^46^ We believe that our presented PAR-DEL methodology can serve as a starting point for approaches that explore high-dimensional activity recording of spatially and temporally controlled cell perturbations, which take advantage of both the power of small molecule pharmacology and the information storage capability of DNA.

## Data availability

The data supporting this article have been included as part of the ESI.†

## Conflicts of interest

There are no conflicts to declare.

## Supplementary Material

CB-OLF-D4CB00137K-s001
