# Supplementary material for: Induced cell phenotype activity recording of DNA-tagged ligands
Source: RSC Chem Biol. 2024 Dec 17;6(2):273–80. doi: 10.1039/d4cb00137k (PMC11701724; doi:10.1039/d4cb00137k)

Supplementary Information (SI) for RSC Chemical Biology.
This journal is © The Royal Society of Chemistry 2025

Induced Cell-based Phenotypic Activity Recording of DNA-encoded Ligands

Philipp N. Sander, Jared T. Gillen Miller, Luke L. Lairson*

Supplementary Material

Table of Contents

Supplementary Material 1
Materials 3
Experimental Methods 3
Plate Coating 3
General Cell Culture 4
Statistical Treatment and Curve Fitting 4
Cell Photoirradiation 4
Transfection 4
Photorelease Studies 5
Microscopy 5
Flow Cytometry 5
PAGE and Western Blot Analysis 6
HaloTag Block 6
Immunoprecipitation 7
qPCR 7
Compound Washout Experiment 7
Synthetic Methods 9
N-Demethyl-Mifepristone 9
6-Amino-hexyl-Mifepristone 10

Mal-PC-6-Amino-hexyl-Mifepristone (Mal-PC-NH_2-MFP) ... 11
Maleic acid-PC-6-Amino-hexyl-Mifepristone (Maleic acid-PC-NH_2-MFP) ... 12
Mal-PC-17-amino-5-androsten-3-ol (Mal-PC-Inactive Control) ... 13
2-(2-(2-(2-azidoethoxy)ethoxy)ethoxy)ethane-1-thiol (N3-PEG-SH) ... 14
DNA Methods and Synthesis ... 15
Plasmid Construction ... 15
DNA Quantification ... 15
Analytical and preparative UHPLC-(ToF-MS) ... 15
Barcode Sequences ... 15
Barcode Assembly ... 17
Barcode Production by PCR ... 18
Double-stranded Barcode Chloroalkane functionalization ... 19
Supplementary Text ... 20
Delivery and Localization ... 20
Supplementary Figures ... 22
Suppl. Scheme 1 ... 23
Suppl. Figure 1 ... 25
Suppl. Figure 2 ... 26
Suppl. Figure 3 ... 27
Suppl. Figure 4 ... 28
Supp. Table 3 ... 29
NMR Spectra ... 30
N-Desmethyl-Mifepristone ... 30
6-Amino-hexyl-Mifepristone ... 32
MS Spectra/LCMS ... 34
Active Barcode: BC1_TC1-PEG-SH ... 34
Active Barcode: BC1_TC1-PC-6-NH_2-hexyl-MFP Fwd ... 35

Active Barcode: BC1_TC1-Chloroalkane Rev 36

Negative Control Barcode: BC1_TC6-PEG-SH Fwd...37

Negative Control Barcode: BC1_TC6-PC-Inactive Control Fwd 38

Negative Control Barcode: BC1_TC6-Chloroalkane Rev 40

Active Barcode: BC1_TC10-PEG-SH 41

Active Barcode: BC1_TC10-PC-6-NH_2-hexyl-MFP Fwd 42

Active Barcode: BC1_TC10-Chloroalkane Rev 43

Normalization Barcode: BC2_TC5 Fwd and Rev -chloroalkane 44

Materials

All chemistry reagents were purchased from Sigma-Aldrich unless further noted. All cell culture reagents were purchased from Gibco, unless further noted.

PCR reagents were purchased from NEB, qPCR reagents from Life Technologies (PowerUp SybrGreen 2X Master Mix).

17-amino-5-androsten-3-ol was purchased as ID 8011-0435 from ChemDiv.

Transfection reagents were procured from Thermo Fisher (Lipofectamine 3000), Polysciences (PEI), Invivogen (LyoVec) and Mirus (Transit-X2).

Experimental Methods

Plate Coating

Polystyrene TC-treated cell culture plates (e.g. for 96 well format: Corning 3595) were treated with 100 µl of Collagen solution in D-PBS (Collagen from calf skin, Sigma C8919, diluted to 0.015% from 0.1% stock concentration) and incubated overnight in a CO2 tissue culture incubator at 37 °C. Collagen solution was removed and plate was washed with 100 ul D-PBS. After plates were dried under laminar air flow, plates were stored in aseptic conditions at 4 °C.

General Cell Culture

HEK293T cells (ATCC) were cultured in DMEM media + 10% heat-inactivated FBS (HI-FBS) + 1% Antibiotic-Antimycotic in a 5% CO_2, 37 °C incubator. Cells were split every 2-3 days using TrypLE. For general experimentation, cells were seeded at a density of 18.75 k cells/200 µl culture media on 96 well plates. Treatments were performed one day after seeding. Cells were regularly tested for mycoplasma contamination.

Statistical Treatment and Curve Fitting

Statistical tests and curve fitting were performed in GraphPad Prism 10. All p values were calculated using an unpaired t test. All samples were performed in technical triplicate replicates unless noted otherwise. For dose response curves, data was normalized to positive and negative controls. Curves were fitted by non-linear regression (least-squares) to a 3-parameter sigmoidal model constrained to 0% and 100% response (defined by negative and positive controls).

Cell Photoirradiation

Cells were seeded at 1750 cells/50 µl per well in white TC-treated 384 well plates (Greiner Bio-One 781080). On the next day, cells were swiftly removed from the incubator and irradiated with lids removed at varying doses of 365 nm UV light (Hitachi F8T5 bulbs) at 4 °C, then returned to cell culture conditions. Viability was measured 48h after irradiation with Cell-TiterGlo luciferase reagent (Promega).

Transfection

For a plasmid+barcode transfection experiment, cells were iteratively transfected with plasmids (in reverse), then barcode (in forward transfection). On day 0, HEK293T cells at <80% confluency were harvested in TrypLE, quenched 1:1 in complete culture media and counted. Cells were diluted to a concentration of 9 million cells/ml in 600 µl complete media in a conical tube. A transfection with 375 ng pSwitch, 3000 ng pGene-HaloTag-GFP using Lipofectamine 3000 was prepared and added to the cell suspension. After 40 min of incubation cells were diluted into a total of 11 ml media, and seeded onto a single 6-well plate. Next day (day 1), cells were harvested, diluted into 8.5 ml media, and seeded onto 96 well plates at 100 µl cell suspension per well. On day 2, cell culture volume was reduced to 40 µl for increased transfection efficiency, and cells were transfected with barcode DNA. After preparation of barcode DNA concentrations, Total DNA dose per well was adjusted to 100 ng with sheared salmon sperm DNA and cells forward

transfected with Lipofectamine 3000 (0.3 µl Lipofectamine 3000 reagent per well). After 4 or 24 hours, wells were washed 2x with 100 µl complete media, optionally UV photoirradiated (see below) and harvested on day 3.

For fluorescently tagged barcode transfections, HEK293T cells seeded to a density of 37500 cells/96 well. Next day, cells were transfected with 100 ng fluorescent DNA barcode according to manufacturer instructions and analyzed the next day.

Photorelease Studies

Prior to photorelease, the CL-1000 UV photo crosslinker with 365 nm bulbs (UVP, Hitachi F8T5 bulbs) was pre-heated in a cold room until power output had stabilized (about 45 min, stable state output of ~ 0.33 J/cm^2 x min). Cells grown on polystyrene cell culture-treated plates (Corning #3598) were transferred to the cold room and the placed in the center of the UV chamber. The plate lid was removed and carefully wrapped in paper tissue and cells were irradiated with 4 J/cm^2. After irradiation the plate lid was replaced, the entire cell culture plate was wrapped in paper tissue and sprayed down with 70% ethanol prior to further processing in a biosafety cabinet or further culturing in an incubator. For barcode transfection experiments, cells were washed twice with 100 µl complete culture media (per 96 well) prior to continued culture.

Microscopy

Cells for microscopy were plated on collagen-coated plates in DMEM Fluorobrite. Prior to imaging, Hoechst 33342 (10 mg/ml, Life Tech) was added at 1:1000 to culture volumes and incubated for 30 min. For TMR-Cl staining, TMR-Cl (Promega) was added to a final concentration of 250 nM to culture media and incubated under culture conditions for 1 hour. Prior to imaging cells were washed with D-PBS with Calcium and Magnesium and subsequently stained for 45 min with Hoechst 33342 (10 nM final concentration) in D-PBS with Calcium and Magnesium. Images were acquired on a Thermo Fisher CX5 CellInsight HCS microscope, 10X objective. Images were processed in the onboard Cell Analysis software to identify nuclei (Hoechst 33342, 386/440 nm excitation/Emission) and quantify GFP (485/521 nm), TMR (560/607 nm), Cy5 (650/694 nm) percent positive and signal intensity.

Flow Cytometry

Cells for flow cytometry were grown on collagen-coated 96-well plates in DMEM media. To prepare for flow cytometry analysis, media was removed, cells were washed gently with D-PBS,

and incubated in 50 µl TrypLE (Gibco) at 37 °C. After addition of 50 µl FACS buffer (2% HI-FBS in D-PBS, 1 mM EDTA, 1% anti-biotic/anti-mycotic), cell layer and clumps were gently broken up by pipetting, and transferred onto a U bottom cell culture plate. After centrifugation at 1200 x rcf, 4 °C for 10 min, supernatant was removed and cells resuspended in 50 µl cold D-PBS with eBioscience™ Fixable Viability Dye eFluor™ 780 (diluted 1:1000, prepared fresh). After 30 minutes of rocking incubation in the dark at 4°C, cells were diluted in 100 µl FACS buffer, resuspended by pipetting, and strained through a 35 µm nylon mesh taped snugly the receiving 96 well plate (flat or U bottom). Cells were gated for singletons and viability (APC-Cy7 channel) on an Agilent Novocyte flow cytometer and analyzed in FlowJo v10.7.

PAGE and Western Blot Analysis

Cells were prepared for PAGE analysis in one of two different workflows: From cells that previously were imaged the D-PBS was removed and cells were treated with cold IP Lysis Buffer (Pierce, with 1X HALT Protease + Phosphatase Inhibitor 50 µl/96 well) on ice. For cells without previous staining for imaging, cells were carefully washed with room temperature D-PBS with Calcium and Magnesium, then treated with cold IP Lysis Buffer on ice. Cells were lysed by orbital shaking and vigorous pipetting. Lysate was used unclarified for further steps. Lysates were denatured in the presence of BOLT LDS Sample Buffer and Bolt Reducing Agent (Thermo Fisher) for 10 min at 70 °C. Samples were separated on BOLT 4-12% gels run in MOPS buffer. Optionally, in-gel fluorescence signal was detected using a BioRad GelDoc MP set to appropriate filter sets, after which proteins were electroblotted onto PVDF membranes (Merck Millipore). Membranes were blocked in 5% skim milk/TBS-T buffer and incubated with primary and secondary antibodies in 5% BSA/TBS-T. Membranes were imaged using a Li-Cor Odyssey gel scanner. Primary antibody for HA epitope: Mouse IgG2a clone F-7 (SCBT), secondary antibody: Goat anti-mouse IgG IRDye 680RD (Li-Cor).

HaloTag Block

To block unliganded HaloTag from reacting with chloroalkane-labeled DNA post-lysis in cell lysis and restricting DNA barcode immunoprecipitation and analysis to DNA inside of activated cells, 7-Bromo-Heptanol (Thermo Fisher) was added to a final concentration of 1 mM to growth media one hour prior to cell harvesting, following Merrill et al.[1]

Immunoprecipitation

Cells were washed with D-PBS with Ca and Mg and lysed on the plate with IP lysis buffer (Pierce) with protease inhibitors and shaken on ice. Unclarified lysate was incubated with anti-HA magnetic beads (covalently attached mouse IgG1 clone 2-2.2.14, Pierce 88836) that were pre- equilibrated with wash buffer (IP lysis buffer with 500 mM NaCl and 100 ng/µl UltraPure Salmon Sperm DNA, Invitrogen). After incubation at room temperature, beads were washed three times with wash buffer. Protein-DNA complexes were eluted for qPCR by incubation in water at 95 °C (10 min) and for protein electrophoresis BOLT loading buffer (Invitrogen) at 70 C° (10 min).

qPCR

DNA barcodes in biological samples were quantified using qPCR with barcode-specific primers and SYBR Green dye. For each biological sample (cell lysate or eluate), 4 technical replicates each were performed. To normalize for lysis yields, immunoprecipitation losses etc., cells were transfected with both a compound-functionalized barcode as well as a non-functionalized barcode that serves as an internal normalization control. Both barcodes are functionalized with Chloroalkane and can be retrieved by HaloTag reaction and immunoprecipitation. Cell lysate or bead eluate were diluted in 1:12 in water, of which 5 ul were added to 10 µl of PowerUp SYBR Green 2X MasterMix (Life Technologies), primers and water to a total volume 20 µl. qPCR was performed on a Biorad CFX384 thermal cycler and analyzed using the CFX manager software. Primer performance was determined using standard dilution curves to ensure PCR efficiencies >95%. Ct values (logspace) were converted to relative barcode amount (linear) using the equation *barcode amount = 2^(-X)*, whereas X is the Ct value. To compare relative barcode amounts between samples, compound-specific relative barcode amounts were normalized using the relative normalization barcode amount.

Compound Washout Experiment

HEK293T cells transfected with pGene and pGene-HaloTag-GFP were seeded at 40% confluency in 100 µl complete media in collagen-coated 96 well plates. Next day, culture media was replaced with 100 µl of media containing 32 µM MFP or DMSO and taken back into culture. After a given amount of time, the culture media was removed, the cells washed with 100 µl media, and finally taken back into culture until 24 hours after initial compound treatment. GFP expression

was determined by flow cytometry. Timepoints assayed were 1, 30, 60, 120, 240, 480, and 1440 minutes.

Synthetic Methods

All solvents and chemicals were of reagent grade. All air- or moisture-sensitive reactions were performed under positive pressure of nitrogen with oven-dried glassware. Unless otherwise specified, commercially available reagents and solvents were used without further purification. Flash column chromatography was carried out on a Teledyne ISCO CombiFlash Rf system using prepacked columns. Analytical-grade solvents (acetonitrile, dichloromethane [DCM], dimethylformamide [DMF], ethanol 99.8% v/v, ethyl acetate [EtOAc], hexane, methanol [MeOH], toluene) were used without further purification. Thin-layer chromatography (TLC) was performed on EMD precoated silica gel 60 F254 plates, and spots were visualized with UV light. NMR experiments were performed with a Bruker Avance III 600 MHz spectrometer. 1H, and 13C NMR data are reported as chemical shifts (δ) in parts per million (ppm) and are calibrated using residual undeuterated solvent as an internal reference. Proton spectra are reported as chemical shift, multiplicity (s = singlet, d = doublet, t = triplet, q = quartet, m = multiplet, and br = broad), coupling constant (J) in Hz, and number of protons. Carbon NMR spectra are reported as chemical shift alone. Yields refer to chromatographically and spectroscopically (1H NMR) homogeneous materials unless otherwise stated. Nominal mass spectra were obtained using an Agilent InfinityLab 1260 mass spectrometer (ESI). Method: A 6 min gradient of 5−95% acetonitrile (containing 0.1% formic acid) in water (containing 0.1% formic acid) was used with a 10 min run time at a flow rate of 1 mL/min. A Zorbax C8 column (5 μm, 4.6 × 50 mm) was used at a temperature of 35 °C. High-resolution mass spectra were acquired on an Waters Xevo G2-XS QToF instrument. All the synthesized target compounds were characterized by 1H NMR, 13C NMR, and/or HRMS and determined to be >95% pure by HPLC analyses.

N-Demethyl-Mifepristone

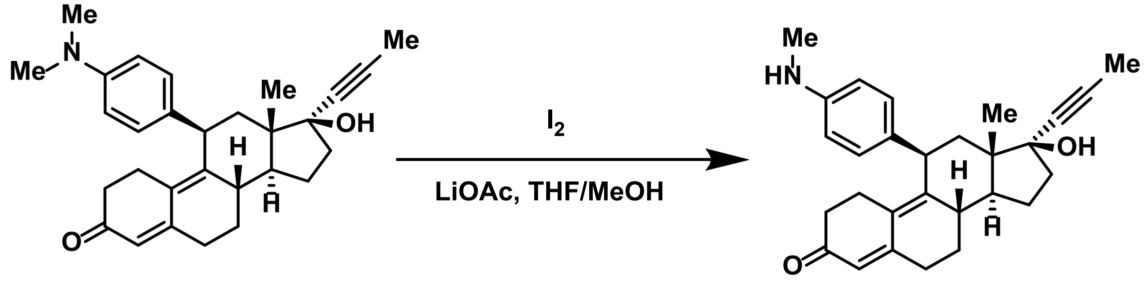

N-Demethyl-Mifepristone/(8S,11R,13S,14S,17R)-17-ethynyl-17-hydroxy-13-methyl-11-(4- (methylamino)phenyl)-1,2,6,7,8,11,12,13,14,15,16,17-dodecahydro-3H- cyclopenta[a]phenanthren-3-one:

Following the procedure published by Wu et al.[2],Iodine (883mg in 8.83 ml methanol) and LiOAc (800 mg) were added under stirring to a solution of Mifepristone in THF (500 mg in 18 ml THF) on ice. The reaction was brought to room temperature and after 3h diluted with EtOAc, washed with H2O and 5% Na2S2O3. The organic phase was washed with water and dried on anhydrous NA2SO4. After filtering solids, the crude reaction mixture was concentrated under reduced pressure at 40°C and loaded onto a silica column. Using a 50:40:10 Hexane:EtOAc:Triethylamine mobile phase material was fractionated and purified to yield a flaky off-yellow substance. ^{1}H NMR NMR (600 MHz, DMSO) δ 6.90 (d, J = 8.3 Hz, 2H), 6.45 (d, J = 8.5 Hz, 2H), 5.65 (s, 1H), 5.42 (q, J = 5.1 Hz, 1H), 5.12 (s, 1H), 4.30 (d, J = 7.1 Hz, 1H), 3.46 – 3.36 (m, 1H), 3.33 – 3.25 (m, 1H), 2.75 (dt, J = 15.5, 5.4 Hz, 1H), 2.63 (d, J = 5.1 Hz, 3H), 2.59 (d, J = 13.9 Hz, 1H), 2.53 (s, 0H), 2.45 – 2.37 (m, 1H), 2.38 – 2.29 (m, 1H), 2.25 – 2.07 (m, 4H), 2.04 – 1.91 (m, 2H), 1.83 (s, 3H), 1.82 – 1.74 (m, 1H), 1.64 – 1.55 (m, 2H), 1.35 – 1.25 (m, 2H), 0.43 (s, 3H).

^{13}C NMR (151 MHz, DMSO) δ 198.34, 157.03, 147.94, 147.63, 132.12, 128.70, 127.78, 122.36, 112.17, 84.62, 80.81, 79.05, 49.90, 46.94, 40.52, 39.50, 39.44, 39.11, 36.86, 30.92, 30.31, 27.77, 25.68, 23.48, 14.11, 3.95.

MS-ESI (m/z): [M+H]+ : calcd for $C_{28}H_{33}NO_2$, 416.3; found, 416.3.

6-Amino-hexyl-Mifepristone

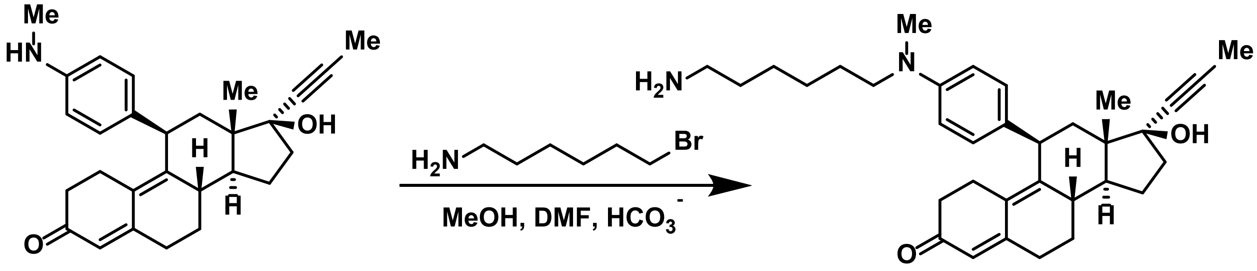

6-Amino-hexyl-Mifepristone/(8S,11R,13S,14S,17S)-11-(4-((6-aminohexyl)(methyl)amino)phenyl)-17-hydroxy-13-methyl-17-(prop-1-yn-1-yl)-1,2,6,7,8,11,12,13,14,15,16,17-dodecahydro-3H-cyclopenta[a]phenanthren-3-one:

Following a procedure modified from Hödl et al.[3], N-Demethyl-Mifepristone (27.7 mg) was dissolved in 660 µl methanol, 200 µl DMF, and 33 µl saturated Sodium Bicarbonate and 6-bromohexan-1-amine (58 mg in 166 µl water) was added dropwise under stirring. The reaction was stirred under exclusion of moisture at 75 °C for 2 hours. Solids were removed by syringe filtration and the reaction mixture concentrated under vacuum. The crude reaction mixture was injected onto a C18 preparative RP-HPLC and purified to yield a flaky off-yellow substance (13 mg, yield: 34.7%). ^{1}H NMR (600 MHz, DMSO) δ 8.41 (s, 3H), 6.97 (d, J = 8.4 Hz, 2H), 6.60 (d, J = 8.5 Hz, 2H), 5.65 (s, 1H), 4.33 (d, J = 7.2 Hz, 1H), 3.27 – 3.21 (m, 2H), 2.83 (s, 3H), 2.80 – 2.69 (m, 3H), 2.63 – 2.53 (m, 2H), 2.43 – 2.30 (m, 2H), 2.27 – 2.06 (m, 5H), 2.04 – 1.93 (m, 2H), 1.83 (s, 3H), 1.83 – 1.75 (m, 1H), 1.65 – 1.43 (m, 8H), 1.35 – 1.15 (m, 12H), 0.41 (s, 3H).

^{13}C NMR (151 MHz, DMSO) δ 198.35, 166.00, 157.01, 147.51, 147.27, 132.03, 128.75, 127.92, 122.39, 112.38, 84.61, 80.83, 79.04, 52.31, 49.90, 46.95, 40.51, 39.44, 39.41, 39.21, 39.10, 38.29, 36.84, 30.92, 28.05, 27.76, 26.55, 26.35, 26.28, 25.71, 23.47, 14.15, 3.95.

MS-ESI (m/z): [M+H]+ : calcd for $C_{34}H_{46}N_2O_2$, 515.4; found, 515.4. HRMS-ESI-ToF (m/z): cald for $C_{34}H_{46}N_2O_2$, 515.3638; found, 515.3643.

Mal-PC-6-Amino-hexyl-Mifepristone (Mal-PC-NH_2-MFP)

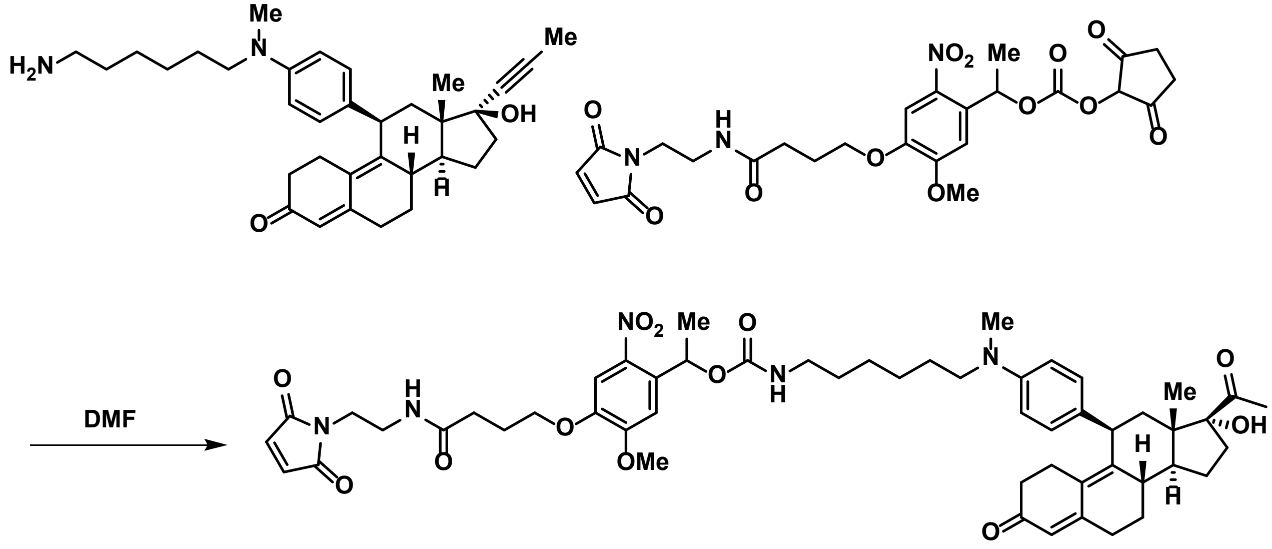

Mal-PC-6-Amino-hexyl-Mifepristone/(1-(4-(4-((2-(2,5-dioxo-2,5-dihydro-1H-pyrrol-1- yl)ethyl)amino)-4-oxobutoxy)-5-methoxy-2-nitrophenyl)ethyl (6-((4-((8S,11R,13S,14S,17S)-17- hydroxy-13-methyl-3-oxo-17-(prop-1-yn-1-yl)-2,3,6,7,8,11,12,13,14,15,16,17-dodecahydro-1H- cyclopenta[a]phenanthren-11-yl)phenyl)(methyl)amino)hexyl)carbamate):

6-Amino-hexyl-Mifepristone (2.7 mg) and 1-(4-(4-((2-(2,5-Dioxo-2,5-dihydro-1H-pyrrol-1-yl)ethyl)amino)-4-oxobutoxy)-5-methoxy-2-nitrophenyl)ethyl (2,5-dioxopyrrolidin-1-yl) carbonate (2.5 mg, “NHS-PC-Mal”, Broadpharm BP-23354) were dissolved in 500 µl dry DMF under argon and set stirring at room temperature. After 70 min, HPLC analysis showed complete conversion of 6-Amino-hexyl-Mifepristone and appearance of both Mal-PC-NH2-MFP and Maleic acid-PC-NH2-MFP (hydrolysis side product of Mal-PC-NH2-MFP). Reaction was purified by preparative C18 RP-HPLC. After lyophilization of fractions, 0.8 mg (15% yield) were recovered. MS-ESI (m/z): [M+H]+ : calcd for $C_{54}H_{67}N_5O_{11}$, 962.5; found, 962.4. HRMS-ESI-ToF (m/z): [M+H]+ : calcd for $C_{54}H_{67}N_5O_{11}$, 962.4915; found, 962.4896.

Maleic acid-PC-6-Amino-hexyl-Mifepristone (Maleic acid-PC-NH_2-MFP)

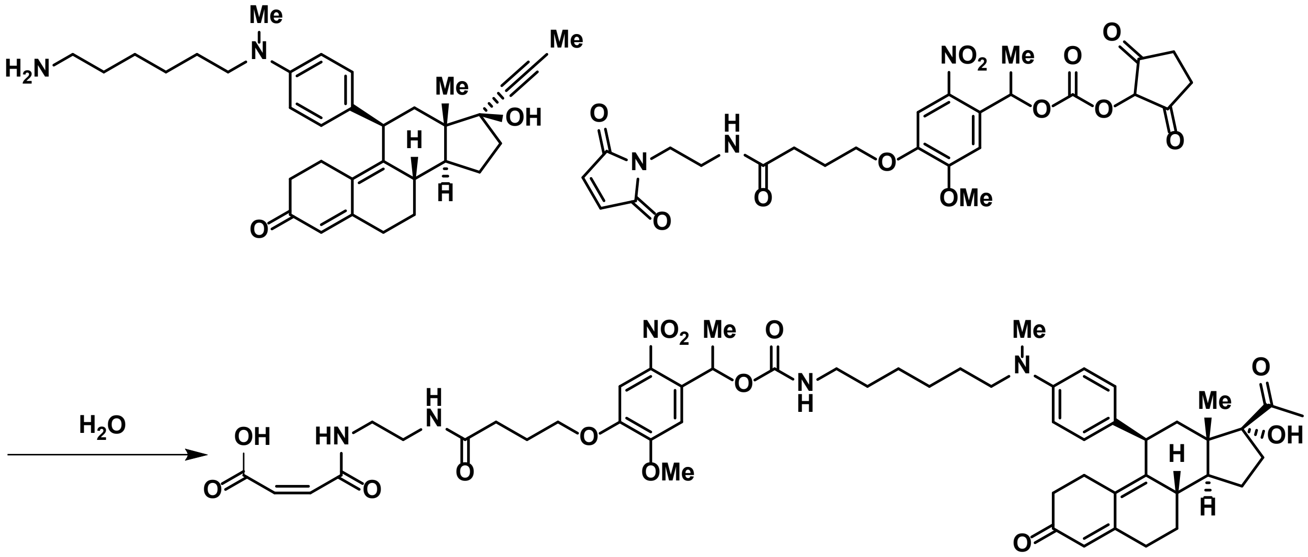

Maleic acid-PC-6-Amino-hexyl-Mifepristone/(Z)-4-((2-(4-(4-(1-(((6-((4-((8S,11R,13S,14S,17S)-17-hydroxy-13-methyl-3-oxo-17-(prop-1-yn-1-yl)-2,3,6,7,8,11,12,13,14,15,16,17-dodecahydro-1H-cyclopenta[a]phenanthren-11-yl)phenyl)(methyl)amino)hexyl)carbamoyl)oxy)ethyl)-2- methoxy-5-nitrophenoxy)butanamido)ethyl)amino)-4-oxobut-2-enoic acid:

Maleic acid-PC-6-Amino-hexyl-Mifepristone was purified from the Mal-PC-6-Amino-hexyl-Mifepristone reaction by RP-HPLC and collected and lyophilized separately. 0.4 mg were recovered. MS-ESI (m/z): [M+H]+ : calcd for $C_{54}H_{69}N_5O_{12}$, 980.5 found, 980.5. HRMS-ESI-ToF (m/z): calcd for $C_{54}H_{69}N_5O_{12}$, 980.5021; found, 980.5089.

Mal-PC-17-amino-5-androsten-3-ol (Mal-PC-Inactive Control)

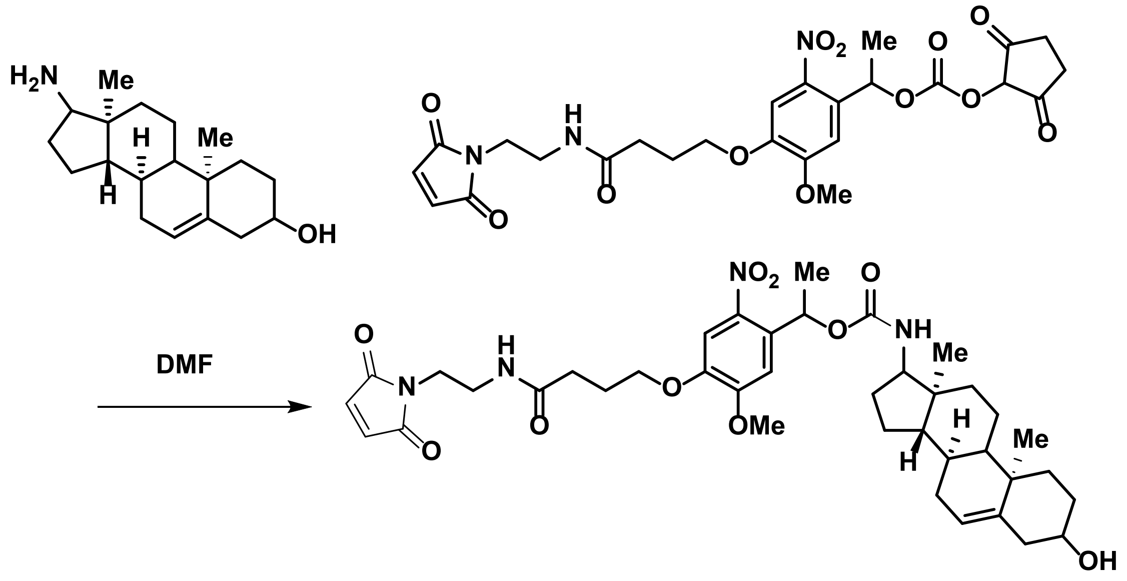

Mal-PC-Inactive Control/1-(4-(4-((2-(2,5-dioxo-2,5-dihydro-1H-pyrrol-1-yl)ethyl)amino)-4-oxobutoxy)-5-methoxy-2-nitrophenyl)ethyl (3-hydroxy-10,13-dimethyl-2,3,4,7,8,9,10,11,12,13,14,15,16,17-tetradecahydro-1H-cyclopenta[a]phenanthren-17-yl)carbamate:

To a solution of 17-amino-10,13-dimethyl-2,3,4,7,8,9,10,11,12,13,14,15,16,17-tetradecahydro-1H-cyclopenta[a]phenanthren-3-ol (“Inactive Control”, 1.00 mg, 3.45 µmol, 1 eq) in DMF (112.5µl), 1-(4-(4-((2-(2,5-dioxo-2,5-dihydro-1H-pyrrol-1-yl)ethyl)amino)-4-oxobutoxy)-5-methoxy-2-nitrophenyl)ethyl (2,5-dioxopyrrolidin-1-yl) carbonate (2.34 mg, 4.16 µmol, 1.20 eq.) followed by TEA (4.82µl, 3.50mg, 34.5µmol, 10 eq.) was added at room temperature and stirred for 15 min. The reaction was monitored by LC/MS and confirmed product formation. The reaction was concentrated to dryness under reduced pressure, then purified by preparative TLC using a 5% MeOH+1% TEA in DCM mobile phase on a 1000µm silica board. A single product was recovered affording an off-white solid (1.23 mg, 48%). TLC (5% MeOH+1% TEA in DCM, Rf): 0.55 (UV). HRMS ESI-ToF (m/z): [M+Na]+, Calcd for $C_{39}H_{52}N_4O_{10}Na$ 759.3581; found 759.3587.

2-(2-(2-(2-azidoethoxy)ethoxy)ethoxy)ethane-1-thiol (N3-PEG-SH)

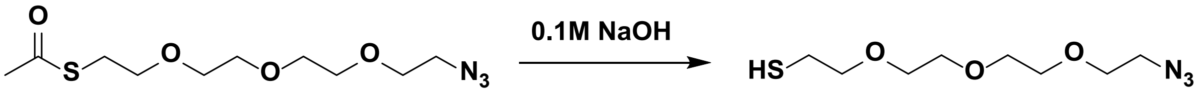

After observed instability of commercially supplied material, 2-(2-(2-(2-azidoethoxy)ethoxy)ethoxy)ethane-1-thiol was prepared fresh for each CuAAC reaction at the micromole scale and immediately used in downstream reactions. S-(2-(2-(2-(2-azidoethoxy)ethoxy)ethoxy)ethyl) ethanethioate (1 μMol: 10 μl of 100 mM in DMSO, Broadpharm BP-20646) was added to 1 μl 1M NaOH for a final concentration of 0.09M NaOH. After 45 min reaction at room temperature or to completion (monitored my LC-MS), the reaction was quenched with 10 μl D-PBS and 1 μl 1M HCl. Reaction progress was determined by MS to be >95%.: MS-ESI (m/z): [M+Na]+ : calcd for $C_8H_{17}N_3O_3S$, 258.0; found, 258.0.

DNA Methods and Synthesis

Plasmid Construction

GeneSwitch plasmids were obtained from Thermo Fisher (K106001). HaloTag-GFP constructs were ordered as gBlocks from IDT and cloned via Gibson Assembly into linearized pGene-A plasmid backbone.

DNA Quantification

DNA was quantified either by 260 nm absorption measurement on a Nanodrop photospectrometer or by polyacrylamide gel electrophoresis and Sybr Gold staining. DNA was mixed 6:1 with 15% Ficoll 400 and loaded onto 8% TBE polyacrylamide gels. After electrophoresis, gels were stained with SyBr Gold, and quantified by densitometry against a DNA standard (single- or double-stranded DNA of the same length).

Analytical and preparative UHPLC-(ToF-MS)

DNA-conjugate synthesis products were analyzed and purified using a Waters Xevo G2-XS QToF instrument. Separation was achieved on a Waters BEH C18 column (mobile phase A: A is 114 mM HFIP/14 mM triethylamine in water, mobile phase B: methanol). Spectra were deconvoluted using MaxEnt1.

Barcode Sequences

DNA Barcodes were constructed from the following two-part combinatorial scheme based on Lyons et al.[4]:

Forward Primer binding site - [AGGGTCTCAGCGTAGCTACGTCCTTTTTGTGCCTCGCTGACGTCGG] - {Batch Code} - common linker [ACGCTA]-{Target Code} - Reverse Primer binding site [TAAATATCTGGAATAGCTCAGAGGC]

where [ ] indicate common sequences and { } indicate variable sequences.

Suppl. Table 1 Barcode Listing

Name	Description	Usage	Forward 5' modification	Reverse 3' modification

BC1_TC1	Active Compound Barcode	In Figure 2	PEG-PC-NH_2-MFP	PEG-chloroalkane
BC1_TC10	Active Compound Barcode	In Figure 4	PEG-PC-NH_2-MFP	PEG-chloroalkane
BC1_TC6	Inactive Control Barcode	In Figure 2,4	PEG-PC-17-amino-5-androsten-3-ol	PEG-chloroalkane
BC2_TC5	Normalization Barcode	In Figure 4	-	PEG-chloroalkane
BC1_TC2	TMR-labeled Barcode	In Supp. Figure 2, 3	(none), TMR labeled on internal T base at position (X)	-OH
BC1_TC8	Cy5-labeled, chloroalkane-labeled Barcode	In Figure 3	Cy5 [IDT]	PEG-chloroalkane

Suppl. Table 2 Barcode Sequences

Name	Sequence
BC1_TC1	AGGGTCTCAGCGTAGCTACGTCCTTTTTGTGCCTCGCTGACGTCGGTAGGCGTCG ATGCCGATCCCGA ACGCTAAATAGCAAGCTGCCTCCACTGTGGAACATAAATATCTGGAATAGCTCAGA GGC
BC1_TC10	AGGGTCTCAGCGTAGCTACGTCCTTTTTGTGCCTCGCTGACGTCGGTAGGCGTCG ATGCCGATCCCGA ACGCTAATTAAGGGCTTACCTTAATGCAGGAACATAAATATCTGGAATAGCTCAGA GGC
BC1_TC6	AGGGTCTCAGCGTAGCTACGTCCTTTTTGTGCCTCGCTGACGTCGGTAGGCGTCG ATGCCGATCCCGA ACGCTAAATAGCAAGCTGCCTCCACTGTGGAACATAAATATCTGGAATAGCTCAGA GGC
BC2_TC5	AGGGTCTCAGCGTAGCTACGTCCTTTTTGTGCCTCGCTGACGTCGGGGATGATAA CCGATACTCGATC ACGCTAATTTAACGACGAAACTTTGCCGGGAACATAAATATCTGGAATAGCTCAGA GGC

BC1_ TC2	AGGGTCTCAGCGTAGCTACGTCCTTTTTGTGCCTCGCTGACGTCGGTAGGCGTCG ATGCCGATCCCGA ACGCTAAACCTCCAAGGTCCCATTCAAGGTTAGATAAATATCTGGAATAGCTCAGA GGC
BC1_ TC8	AGGGTCTCAGCGTAGCTACGTCCTTTTTGTGCCTCGCTGACGTCGGTAGGCGTCG ATGCCGATCCCGA ACGCTAATAATGCCACCGAGTTCCCACAGGAACATAAATATCTGGAATAGCTCAGA GGC

Barcode Assembly

Barcodes functionalized with photoreleaseable compounds were constructed following the workflow shown in Suppl. Scheme 1. Replace NH_2-MFP with 17-amino-5-androsten-3-ol for inactive control barcode (BC1_TC6). Single-stranded DNA oligos functionalized with either a 5' hexynyl or a 5' amino modifier C6 were ordered from IDT.

Following numbering in Suppl. Scheme 1:

1) To produce compound-functionalized Forward strand, 5' hexynyl Oligo (127 nt) was reacted using CuAAC chemistry with freshly synthesized N3-PEG-SH. Reaction was prepared by adding reagents in the following order: 300 µl DNA (200 µM), 40 µl D-PBS with Calcium and Magnesium, 20 µl of freshly prepared N3-PEG-SH solution (~50 mM, see chemical synthesis section), 40 µl catalyst solution. Catalyst solution was prepared fresh from THPTA (10 mM), $CuSO_4$ (10 mM) and freshly prepared Sodium Ascorbate (10 mM). A high CuSO4 concentration was chosen to increase reaction rate and saturate thiol-copper chelation effects. Reaction was set at 37 °C for 4 hours or until completion, as monitored by UHPLC-ToF-MS. Reaction was quenched with addition of EDTA to a a final concentration of 10 mM. Reaction was purified by ethanol precipitation: 3M Sodium Acetate solution (pH 5.2) was added to quenched reaction to a final concentration of 0.3M. EtOH (100%, stored at -20) was added to the reaction mixture in a 2.5:1 volume ratio and the reaction tube set at -20 °C overnight. Next day, the precipitated DNA was pelleted at 20,000 x rcf, - 11° C for 1 hour in a precooled centrifuge. The pellet was washed twice with 70% EtOH with 10 mM EDTA and pelleted for 20 min. After the final centrifugation step, the wash solution was removed and the remaining EtOH dried at room temperature under reduce atmosphere in a SpeedVac. To resuspend the pellet, 50 µl of ddH2O were

added and the pellet incubated at 37 °C with regular vortexing. Delays in processing the ethanol precipitated DNA after reaction completion led to an increase in blue color in the pellet that correlated with a reduced yield of intact DNA. DNA concentration was

determined by A260 absorption using a Nanodrop. Reaction success was assessed by UHPLC-ToF-MS.

2) Thiol-functionalized DNA (~X µM) was reduced with a freshly prepared 5 mM TCEP working solution buffered to ~pH 7.5 (5 µl of freshly prepared 100 mM TCEP solution, 17.5 µl 0.1M NaOH, 77.5 µl 0.1M Tris pH 8) in a 1:1 volume ratio for 10 min. After that, an additional volume equivalent of steroid-coupled photorelease maleimide (e.g. Mal-PC-NH_2-MFP, 10 mM in DMSO) was added. Reaction proceeded at room temperature in the dark and was ran until completion (up to 30 min), as monitored by UHPLC-ToF-MS. Steroid functionalized DNA experienced a strong shift in retention on C18 reverse phase UHPLC-MS, and was purified by fraction collection of repeat injections of crude reaction mixture onto the analytical UHPLC column. Fractions were pooled, flash-frozen and lyophilized. DNA pellets were resuspended in ddH2O, submitted for purity determination by UHPLC-MS and quantified both by Nanodrop and SyBr Gold gel staining.
3) 5' Amino modifier C6 reverse strand oligo (200 µM, 20 µl) was adjusted to pH 8.5 with 1 µl Sodium Borate Buffer (0.091M) and then speedvacced to a pellet. Pellet was resuspended in 2 µl water, and 10 µl of NHS ester-choloroalkane (5 mg/ml in DMSO, Promega P6751) were added. Reaction progressed at 37 °C for at least 90 min or until completion as determined by UHPLC-ToF-MS. Reactions with 95% purity were Ethanol precipitated (see 1)). If the reaction failed to reach completion, crude reaction mixture was injected onto the analytical UHPLC column and fractions were collected. Fractions were pooled, flash-frozen and lyophilized. DNA pellets were resuspended in ddH2O, submitted for purity determination by UHPLC-MS and quantified both by Nanodrop and SyBr Gold gel staining.
4) To prepare doublestranded DNA barcodes, equimolar amounts of compound-functionalized forward strand and chloroalkane-functionalized reverse strand were added to ddH2O, adjusted to a concentration of 20 ng DNA/µl and then hybridized by incubating for 30 s at 98 °C and then gradually cooling to room temperature. This working stock was subsequently used for cell transfection experiments.

Barcode Production by PCR

Fluorescently labeled barcodes (BC1_TC2, BC1_TC8) were produced by large-scale PCR amplification (Phusion polymerase, NEB) using standard programs of a gBlock template (IDT) with a fluorescently labeled Fwd primer (IDT) and a chloroalkane-functionalized Rev primer (Ordered as 5' Amino modifier C6 from IDT, then functionalized according to step 3) described in

barcode assembly. PCR reactions (800-1600 µl reaction volume) were pooled and purified using a Monarch® PCR & DNA Cleanup Kit (NEB) and quantified on a nanodrop spectrophotometer using both A260 and fluorophore peak absorption wavelengths.

Double-stranded Barcode Chloroalkane functionalization

BC2_T5 was ordered at 5' OH Fwd oligo and as 5' Amino modifier C6 Rev oligo (both 127 nt, from IDT). Oligos were prepared to a final concentration of 100 µM and hybridized by incubating for 30 s at 98 °C and then gradually cooling to room temperature. Hybridized DNA (200 µM, 20 µl) was adjusted to pH 8.5 with 1 µl Sodium Borate Buffer (0.091M) and then speedvacced to a pellet. Pellet was resuspended in 2 µl water, and 10 µl of NHS ester-choloroalkane (5 mg/ml in DMSO, Promega P6751) were added. Reaction progressed at 37 °C for at least 90 min or until completion as determined by UHPLC-ToF-MS. Reactions with 95% purity were Ethanol precipitated (see 1)).DNA pellets were resuspended in ddH2O, submitted for purity determination by UHPLC-MS and quantified by Nanodrop.

Supplementary Text

Delivery and Localization

We studied and optimized delivery of functionalized DNA into cells to identify optimal assay conditions. The field of nucleic acid transfection has focused on two general types of nucleic acid payloads: large, double-stranded circular DNA, such as plasmids, or short single-stranded linear RNA, such as siRNA. Thus, reagents and protocols are optimized for delivery for one or both payload archetypes. To evaluate the suitability of different commercial transfection reagents to deliver a short, linear DNA barcode, performed an exploratory screen of transfection reagents, including Lipofectamine 3000, Polyethyleneimine (PEI) (Supp. Fig. 2A), Transit-X2 and LyoVec (Supp. Fig. 3), representing various types and generations of polymeric and lipid nanoparticle transfection technology. We transfected a rhodamine fluorophore (TMR)-labeled 127 bp DNA barcode into HEK293T cells and washed and imaged 24h after transfection. Turning our attention to the most commonly used transfection reagents Lipofectamine 3000 and PEI, Lipofectamine 3000 showed stronger intracellular transfected DNA barcode signal compared to PEI, with more of punctae present suggesting that remaining transfection complexes were clinging to the culture vessel (Supp. Fig. 2A). We reasoned that delivery to the nucleus might be possible and could be advantageous due to the existence of DNA-sensing innate immunity pathways in the cytosol that unduly stress and skew a phenotypic assay. We compared the relative staining pattern of the TMR DNA barcode signal with the cell nucleus staining (with Hoechst 33342) to gauge if the DNA barcode is delivered to the nucleus, or is retained in the cytosol. Interestingly, for both transfection reagents the staining patterns suggest a predominantly cytosolic delivery, with cells often showing a clear nuclear “shadow” in the TMR stain that is complementary to the Hoechst nuclear DNA stain, with white arrows showing the nucleus and correspondent lack of TMR signal in illustrated in Figure 2A. In the search for differences in localization and transfection efficiency as well as to study the kinetics of delivery we extended our investigation to a broader panel of transfection conditions using PEI at a higher N:P ratio (previously 3, now 30) and Lipofectamine 3000 at different time points and vigorously washing away excess transfection reagent/DNA complexes after either 4h or 24h contact with cells. DNA delivery efficiency was measured by flow cytometry 24h post transfection to detect the TMR-labeled 127 bp DNA barcode (Supp. Fig. 2B). Although PEI (at a very high N:P ratio of 30) led to higher intracellular DNA signal after 4h when compared to Lipofectamine 3000, Lipofectamine 3000 showed an 8.7x higher transfection efficacy after 24h of cell contact as compared to 4h of cell contact. The staining intensity was equivalent to PEI at

24h, but showed reduced impact on apparent cell viability as evident by a higher number of stained nuclei (Supp. Fig. 2C, 3). This suggests that 4h of cell contact is sufficient to deliver an appreciable amount of DNA barcodes while maintaining high cell viability. Utilizing short contact times of cells with transfection reagents can be useful to by allowing both high transfection in a high cell density setting, and experiments in low-density cell culturing conditions by with subsequent trypsination and re-seeding. As cell lines are known to show different responses to drugs at different cell densities[5], being able to use short transfection times makes the PARDEL methodology more broadly applicable to different experimental settings.

Supplementary Figures

Suppl. Scheme 1

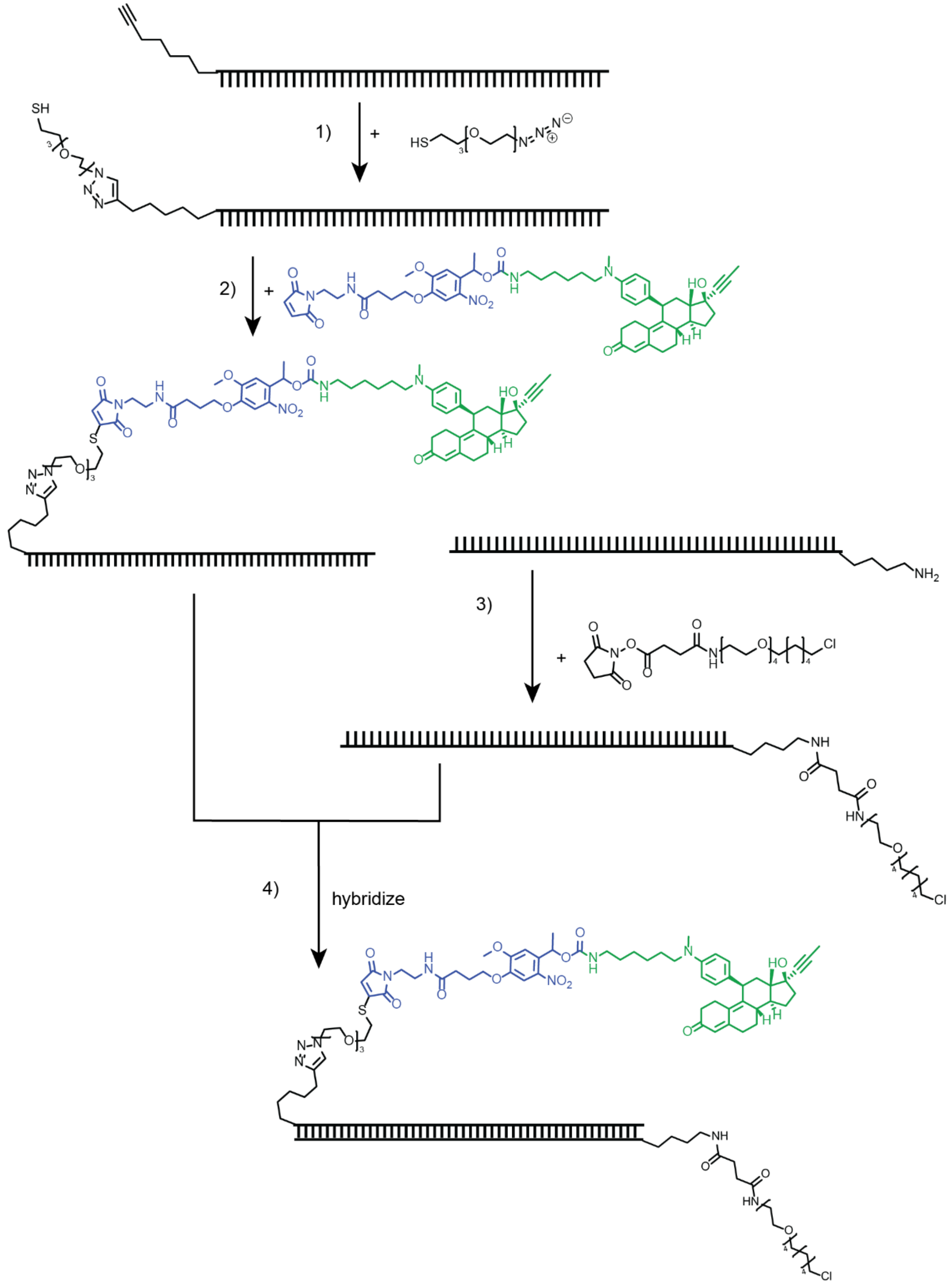

Legend:

Scheme of assembly process of compound-functionalized DNA barcodes, exemplified with 6-Amino-hexyl-MFP (green). For detailed description, see “Barcode Assembly” methods description.

Suppl. Figure 1

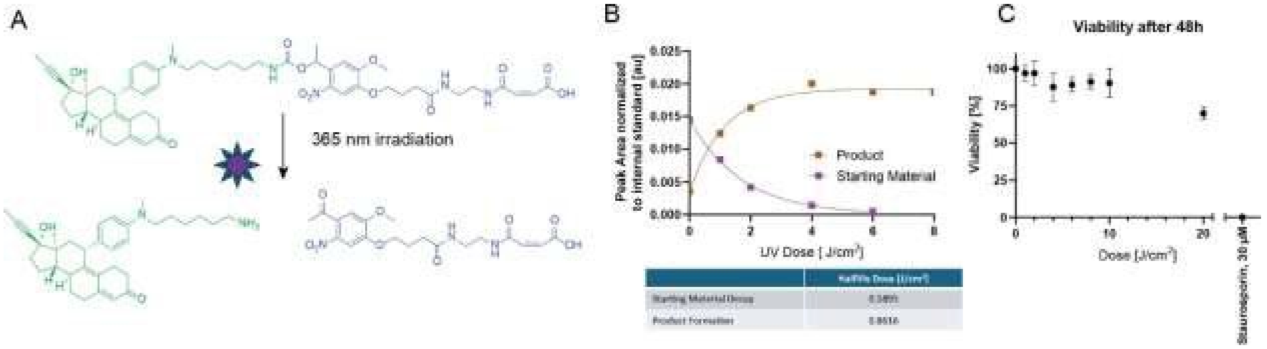

Legend:

A) Scheme of model photorelease reaction. B) Quantification of remaining Starting Material (Maleic Acid-PC-NH_2-MFP) and formed Product (NH_2-MFP) tracked by LC-MS, quantified against an internal standard, n=1. C) Viability of HEK293T cells measured 48 hours after 365 nm irradiation with varying doses. As a control for cell death, control cells were treated with 30 µM Staurosporin.

Suppl. Figure 2

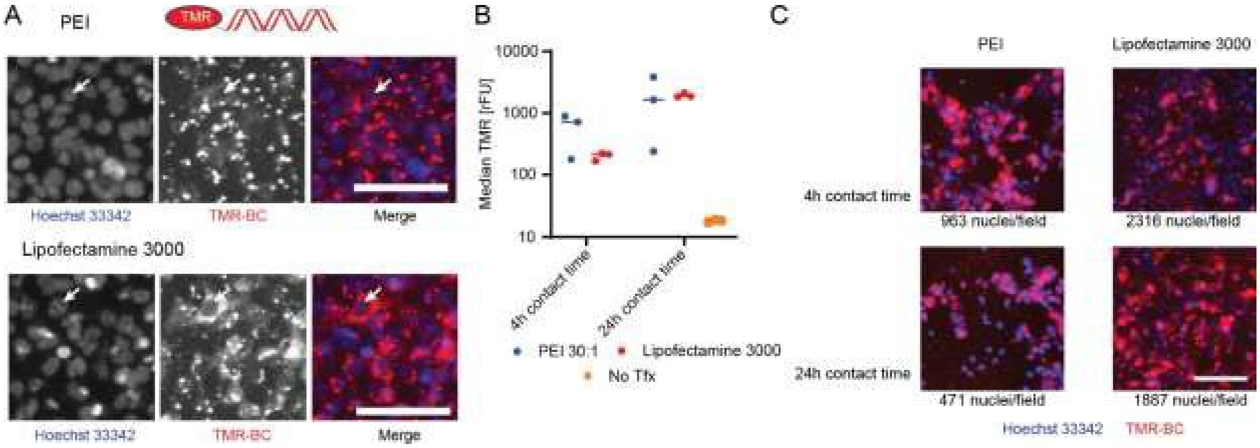

Legend:

PEI or Lipofectamine-3000 mediated delivery of TMR-labeled Barcode DNA to HEK293T cells imaged after 24h. Scale bar equivalent to 100 µm. (B) Flow cytometry quantification and (C) enlargement from representative fluorescence microscopy images of DNA delivery of TMR-labeled Barcode DNA to HEK293T cells harvested 24h after transfection, testing contact time and transfection reagent. Cells were exposed to TMR-labeled DNA in transfection complexes (PEI or Lipofectamine 3000) for 4 or 24 h of contact time. Number of nuclei counted in entire imaging field shown below the images. Scale bar equivalent to 100 µm.

Suppl. Figure 3

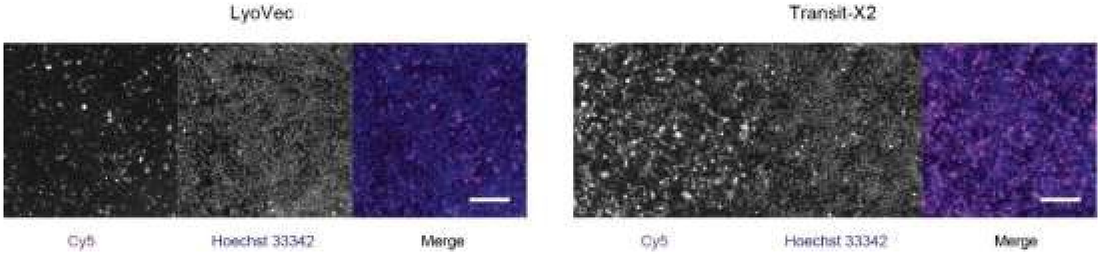

Legend:

HEK293T cells transfected with Cy5-labeled DNA barcode using either LyoVec or Transit-X2 and imaged 24 hours after transfection. Scale bar equivalent to 100 μm.

Suppl. Figure 4

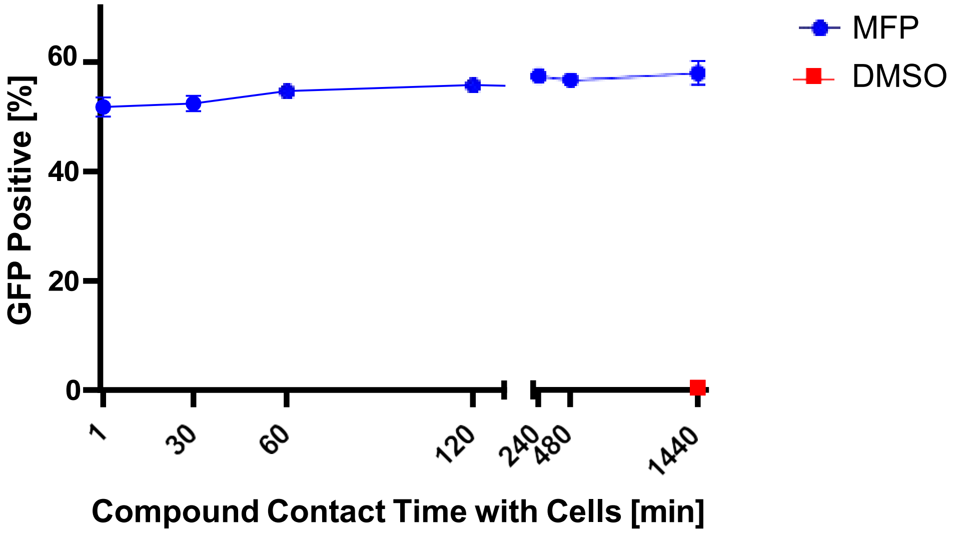

Legend: GFP expression as a function of contact time of 32 µM MFP or DMSO with pSwitch/pGene-HaloTag-GFP expressing HEK293T cells, n = 3.

Suppl. Table 3

Main Text Figure	Assay	Comparison	Estimated EC_X	Z' Factor
2D	GFP Fluorescence	Positive to Negative Control	100	0.92
4B	GFP Fluorescence	Positive to Negative Control	100	0.94
4B	GFP Fluorescence	Active to Inactive Compound with transfection reagent and UV treatment	20	0.78
4C	DNA Recovery	Active to Inactive Compound with transfection reagent and UV treatment	20	0.21

Legend:

Z' Factor calculation for select data comparisons for data shown in main text figures. Estimated EC_X is an estimation of effective concentration, where X is the percentage of maximum signal.

NMR Spectra

N-Desmethyl-Mifepristone

^1H, 600 MHz, DMSO-d6

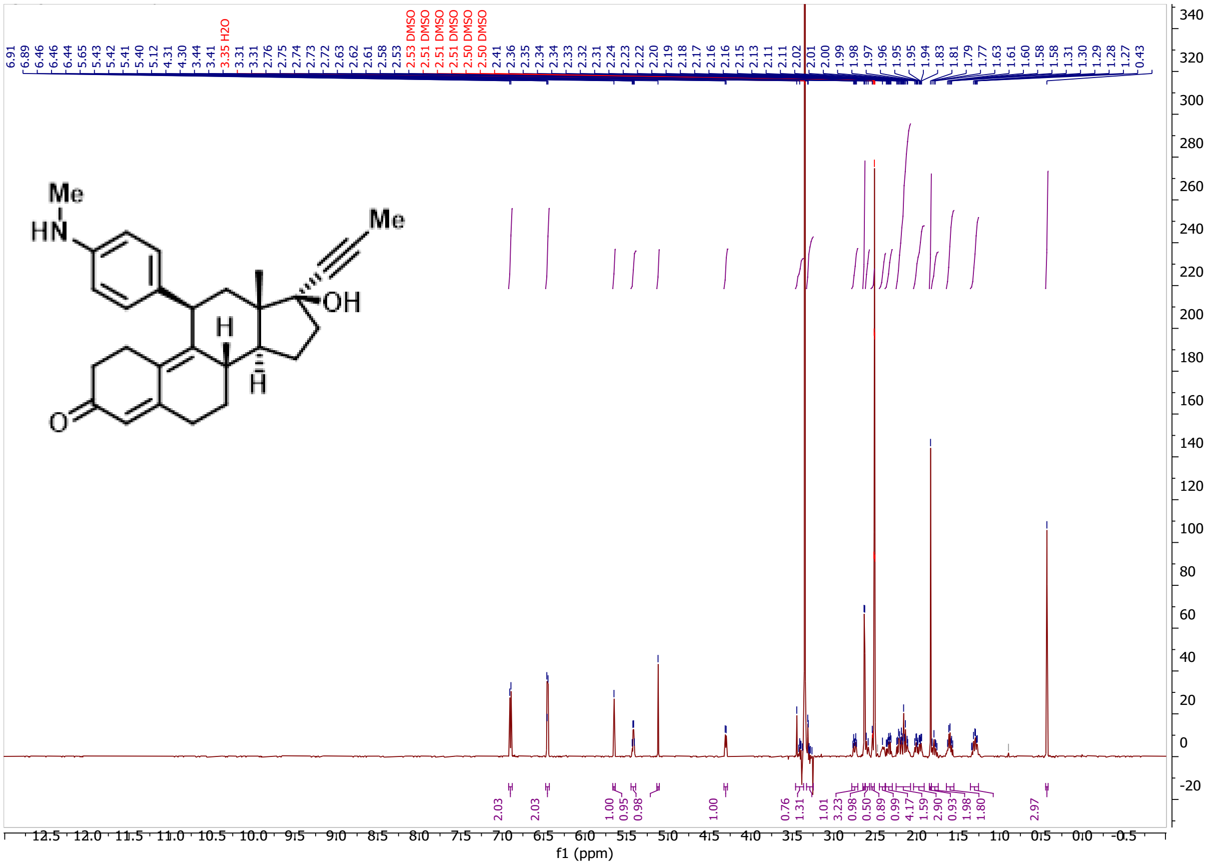

^{13}C, 150 MHz, DMSO-d6

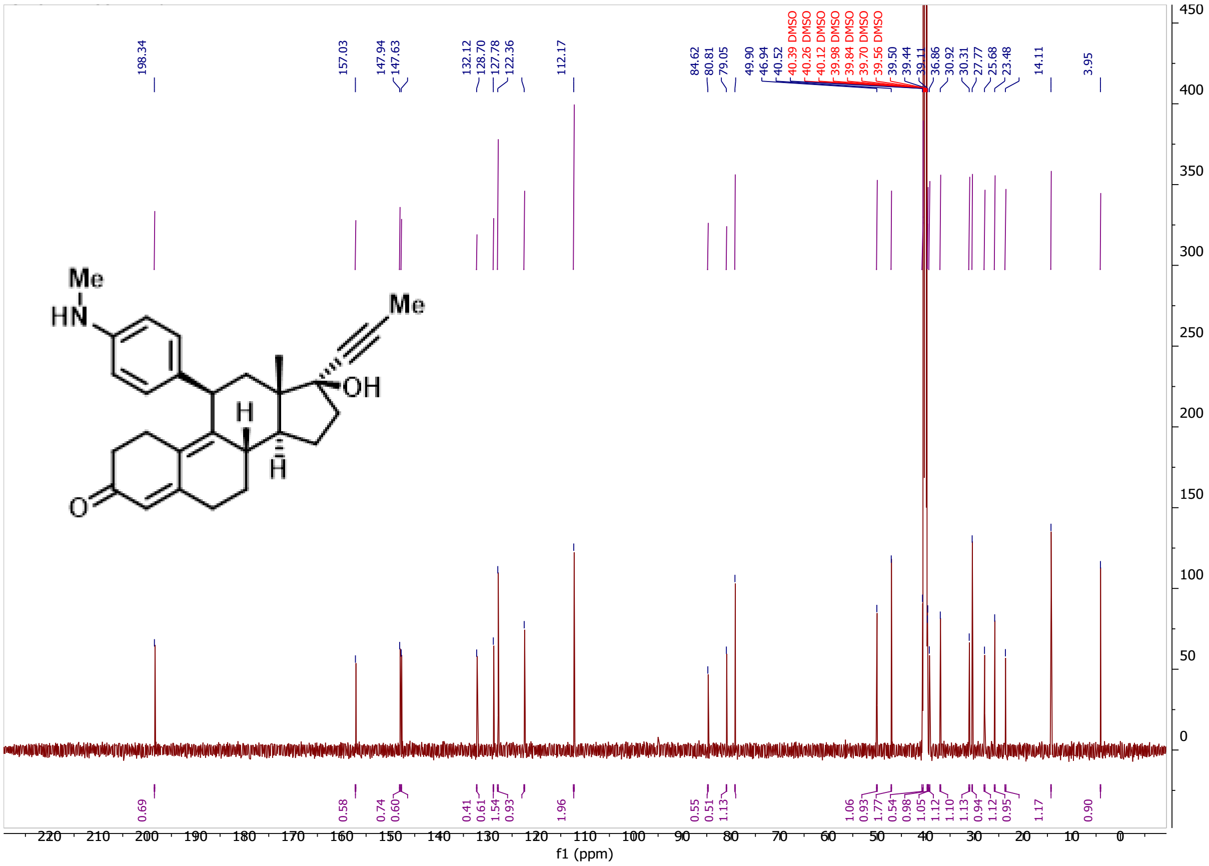

6-Amino-hexyl-Mifepristone

^{1}H, 600 MHz, DMSO-d6

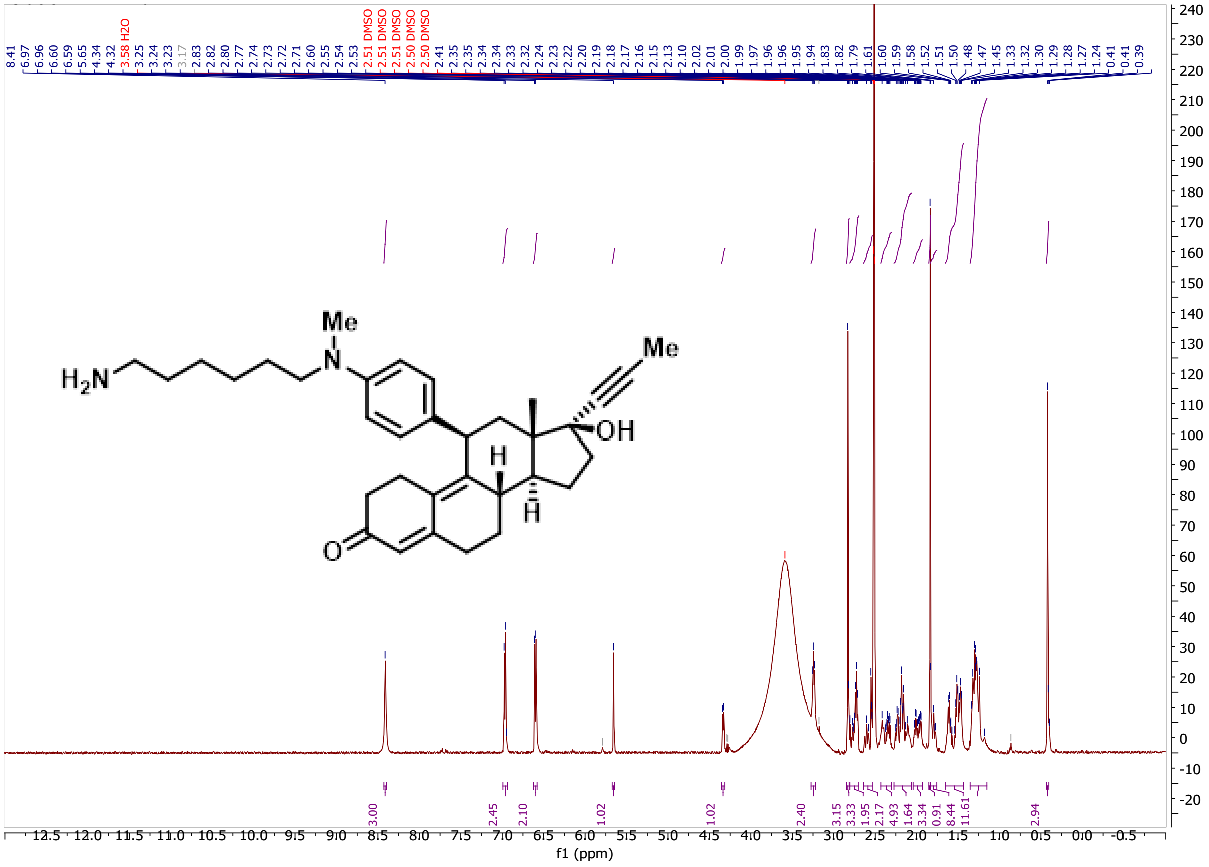

^{13}C, 150 MHz, DMSO-d6

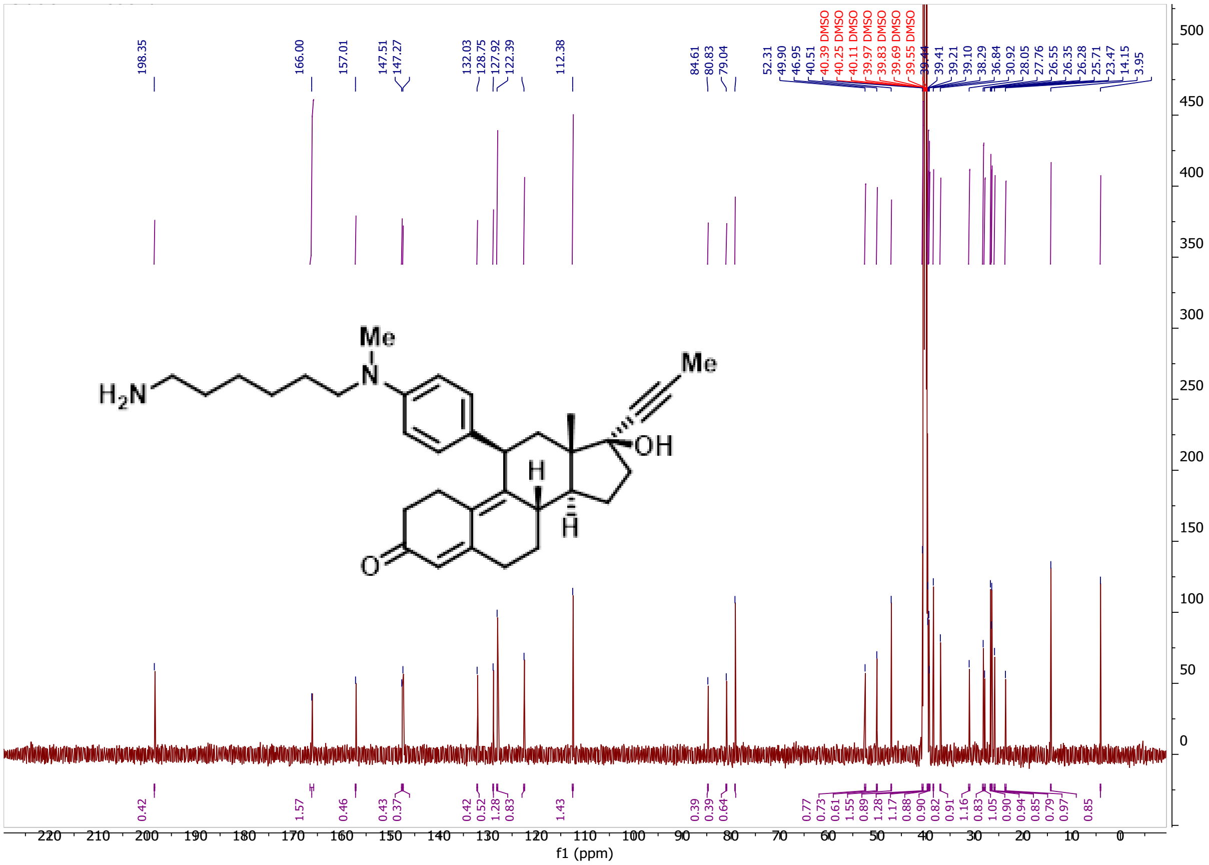

MS Spectra/LCMS

Active Barcode: BC1_TC1-PEG-SH

Deconvoluted Peak. Expected mass: 39567, detected mass: 39566. 39171 is side product from commercial synthesis.

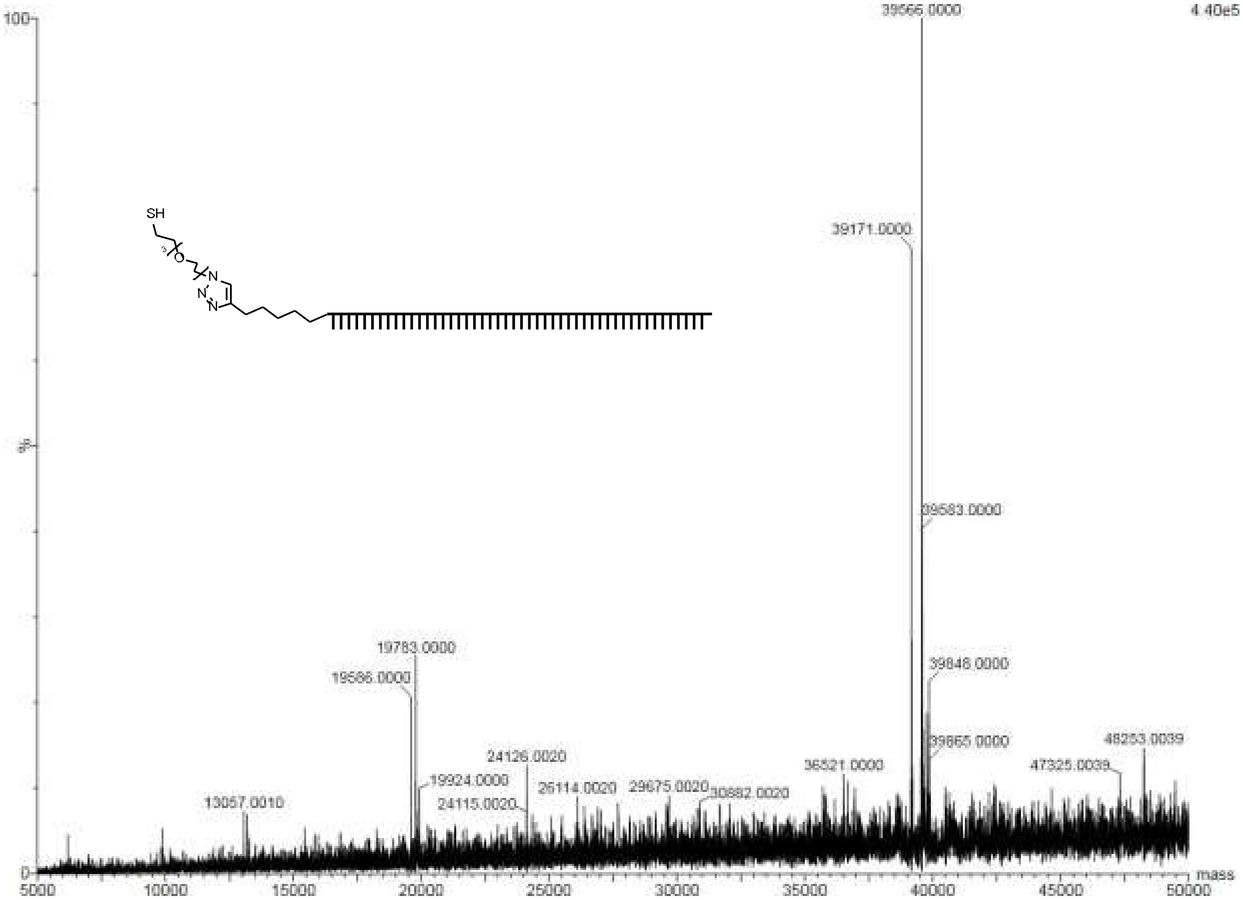

Active Barcode: BC1_TC1-PC-6-NH_2-hexyl-MFP Fwd

TIC Chromatogram

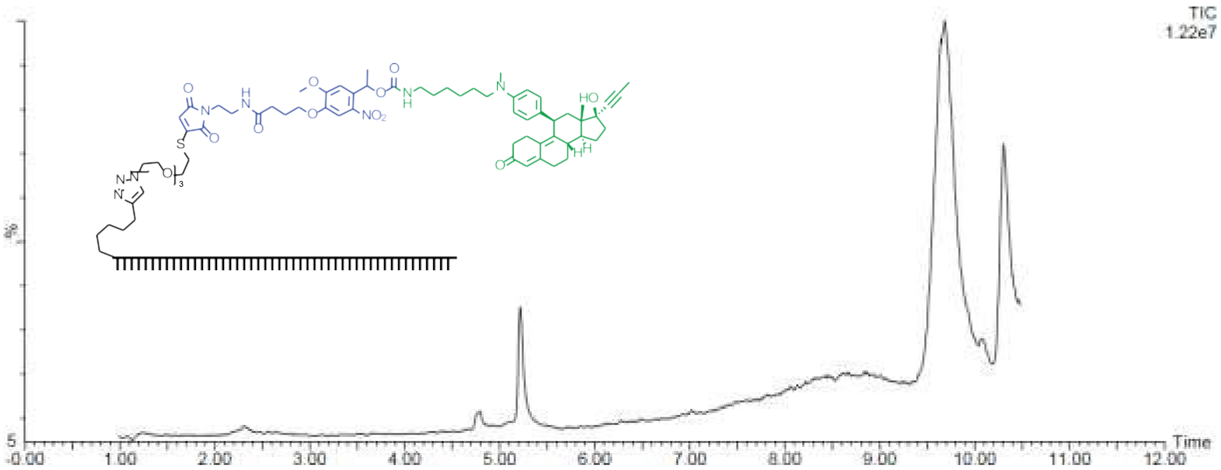

Deconvoluted Peak @ 5.2 min. Expected mass: 40530, detected mass: 30529

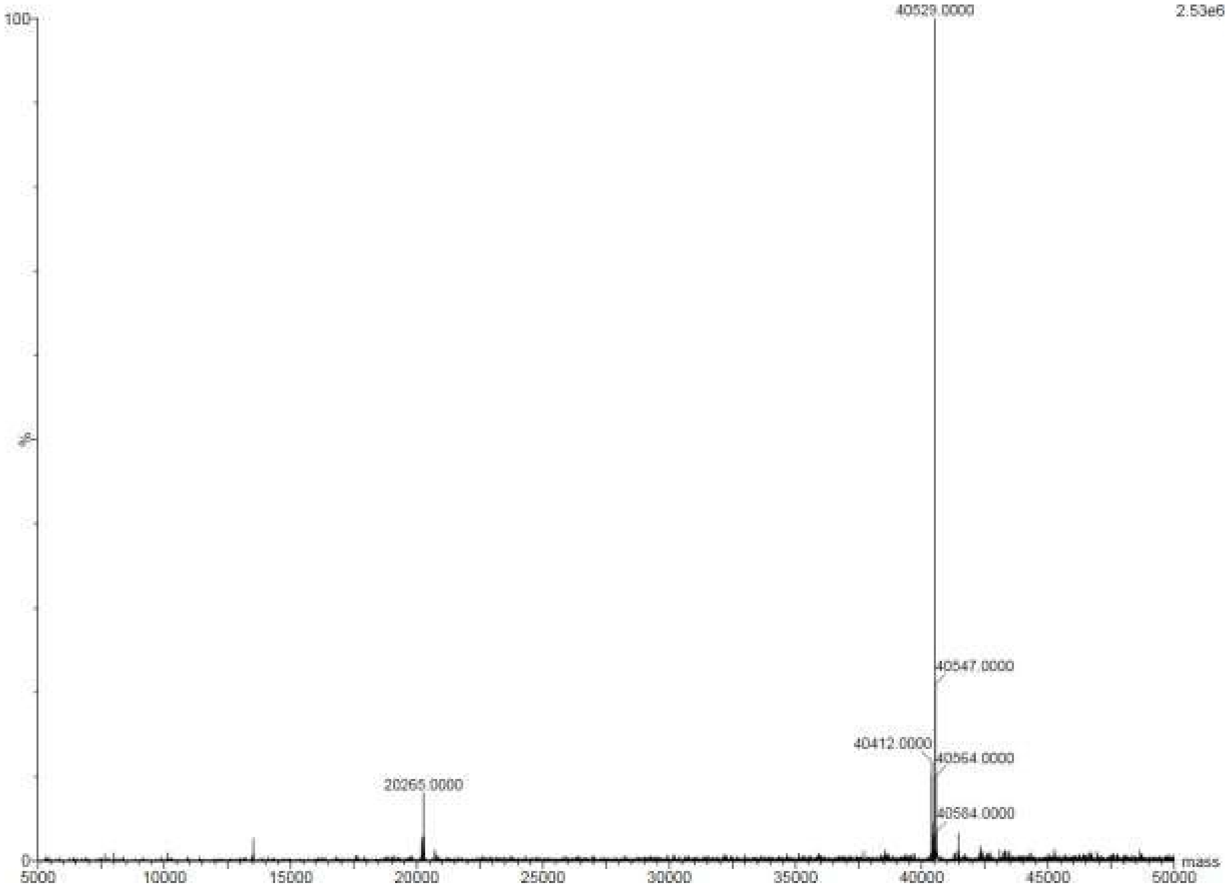

Active Barcode: BC1_TC1-Chloroalkane Rev

TIC Chromatogram

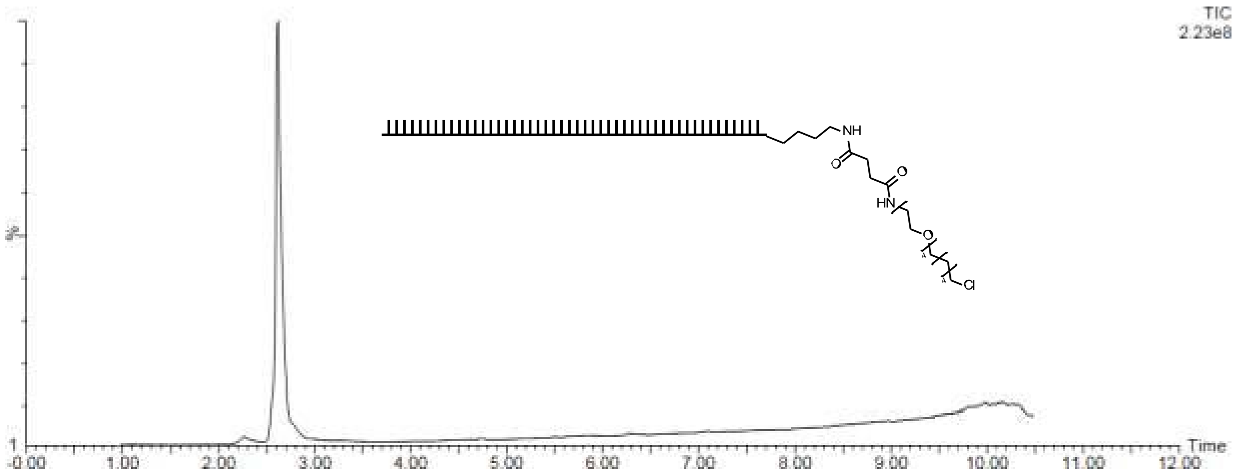

Deconvoluted Peak @ 2.6 min. Expected mass: 39754, detected mass: 39759

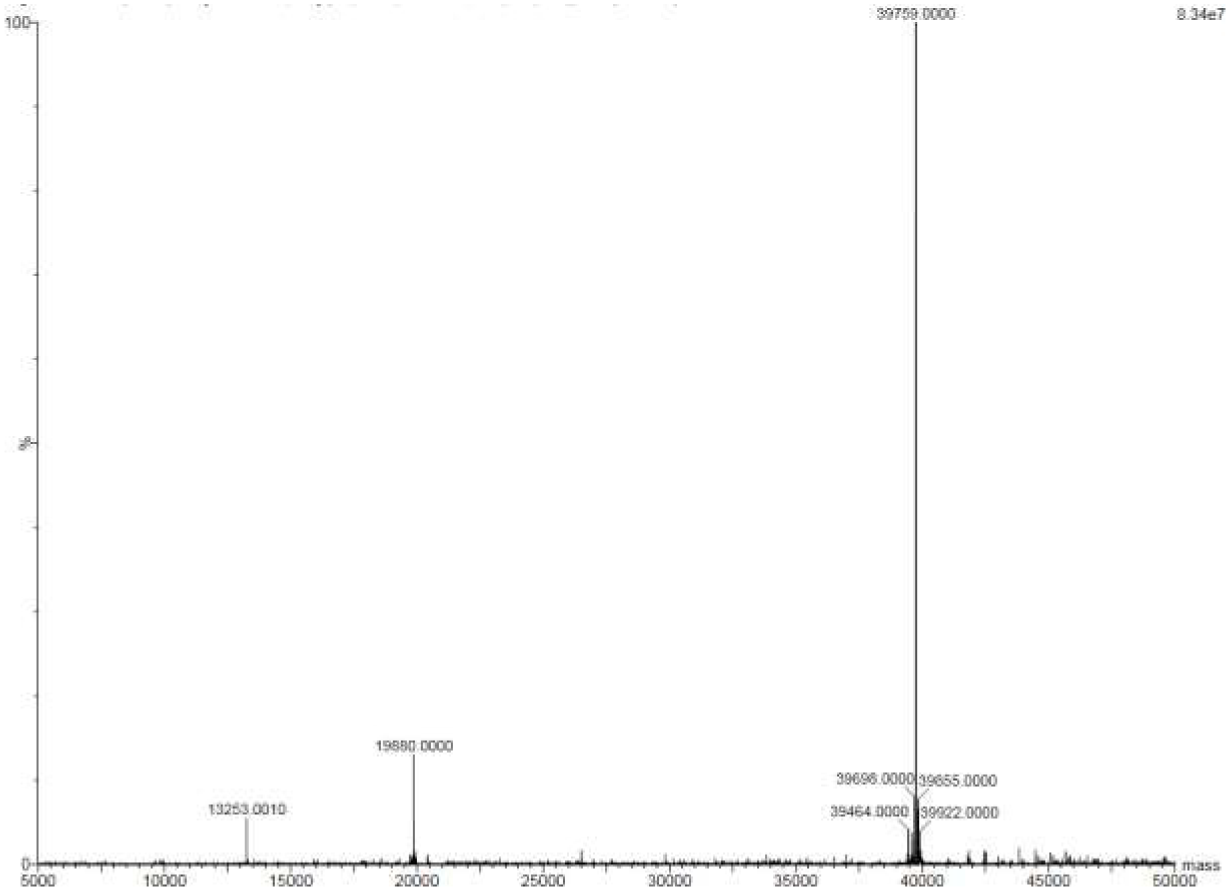

Negative Control Barcode: BC1_TC6-PEG-SH Fwd

Deconvoluted Peak. Expected mass: 39622, detected mass: 39621.

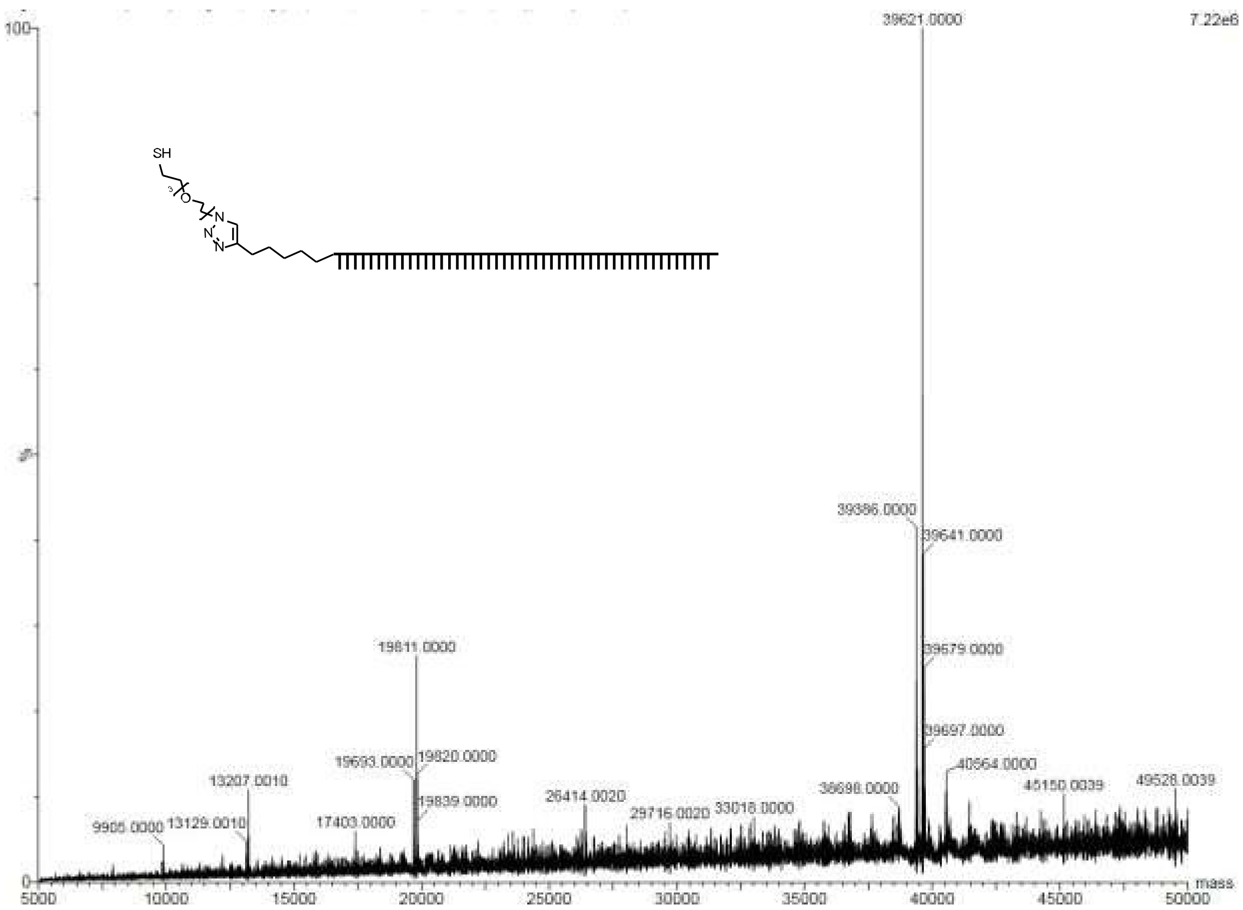

Negative Control Barcode: BC1_TC6-PC-Inactive Control Fwd

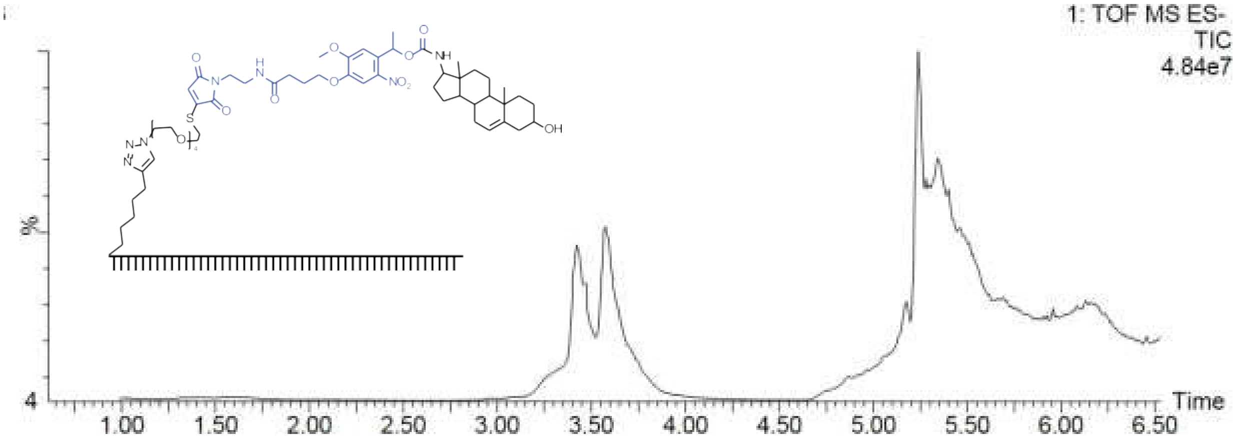

Deconvoluted peak at 3.4 min. Expected mass: 40358, detected: 40358.

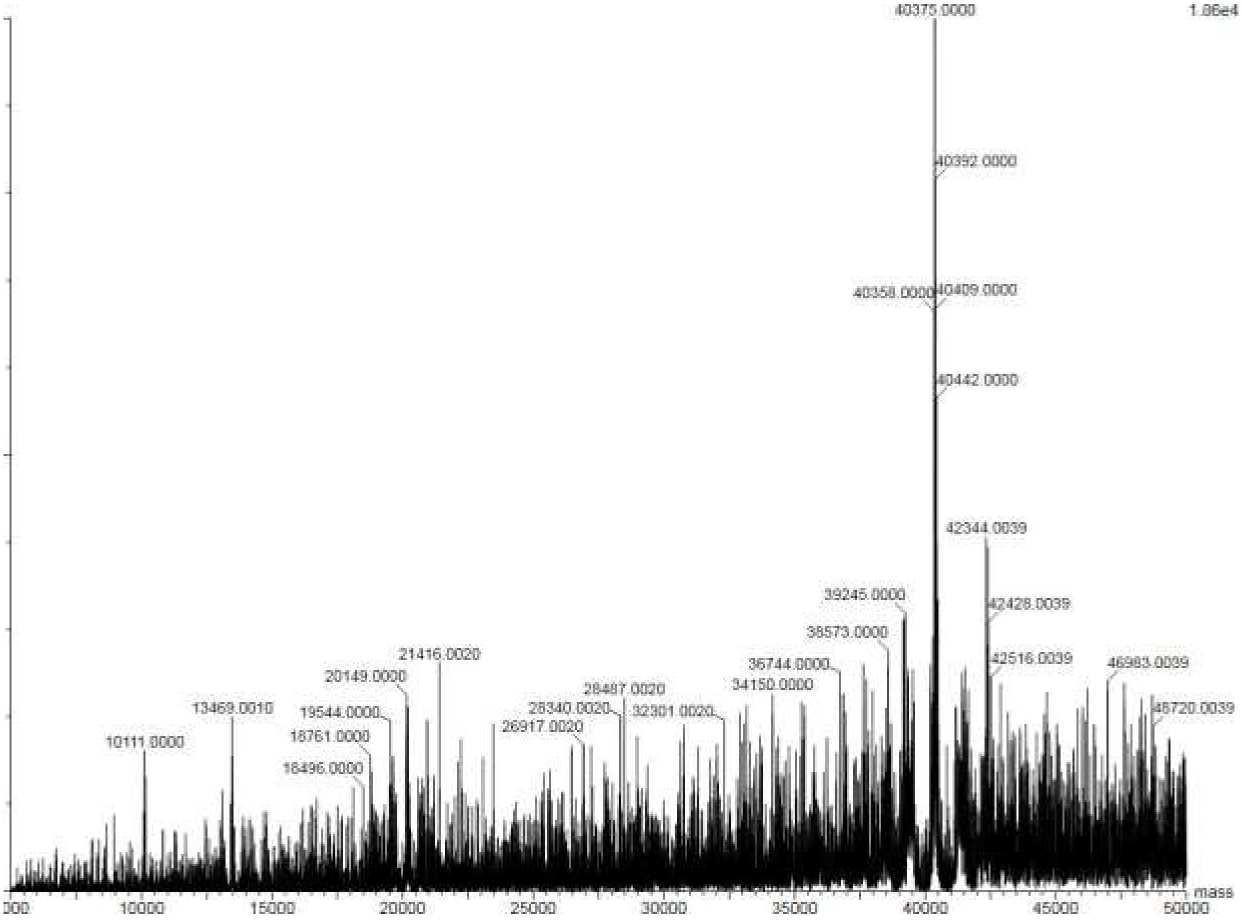

Deconvoluted peak at 3.8 min. Expected mass: 40358, detected: 40357.

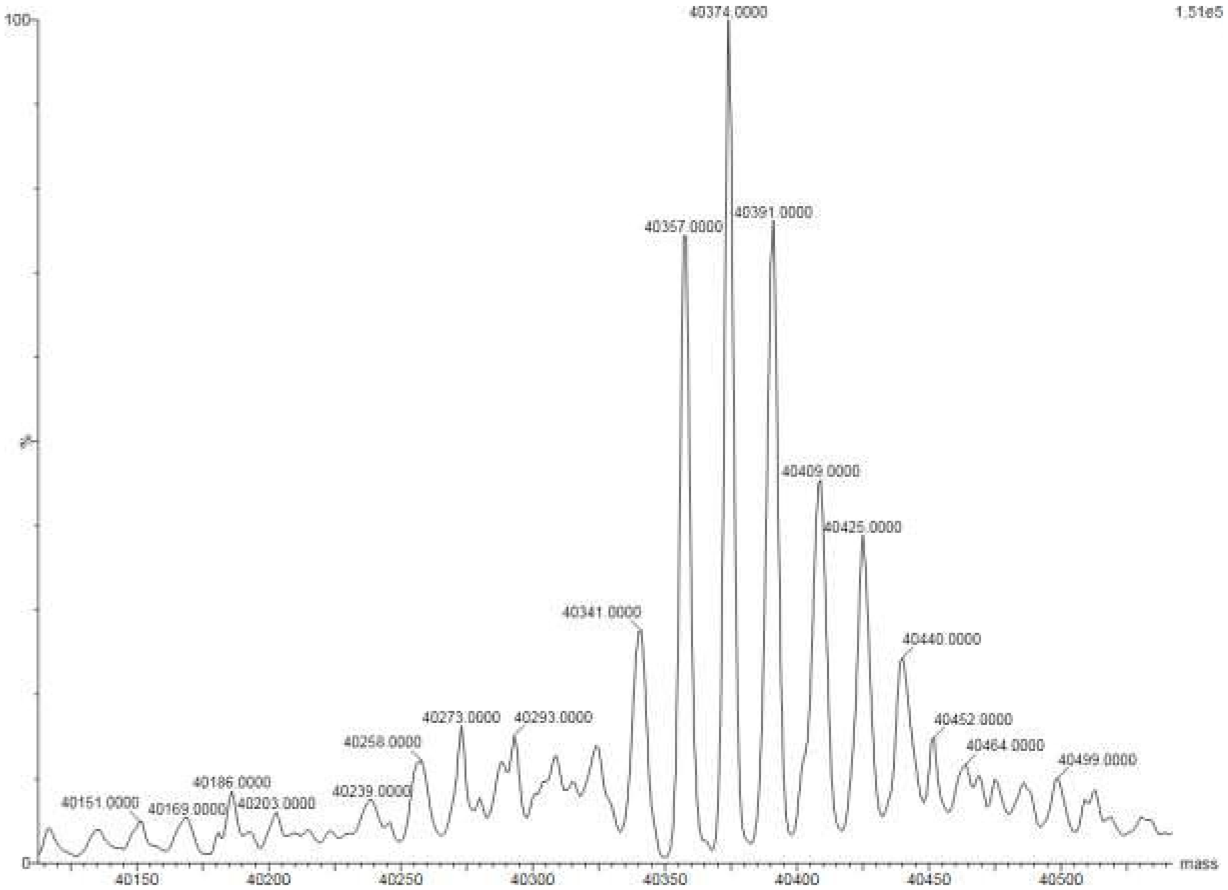

100
%
0
1.51e5
40374.0000
40357.0000
40391.0000
40409.0000
40425.0000
40341.0000
40440.0000
40452.0000
40464.0000
40499.0000
40273.0000
40293.0000
40258.0000
40239.0000
40186.0000
40151.0000
40169.0000
40203.0000
40150
40200
40250
40300
40350
40400
40450
40500
mass

Negative Control Barcode: BC1_TC6-Chloroalkane Rev

TIC Chromatogram

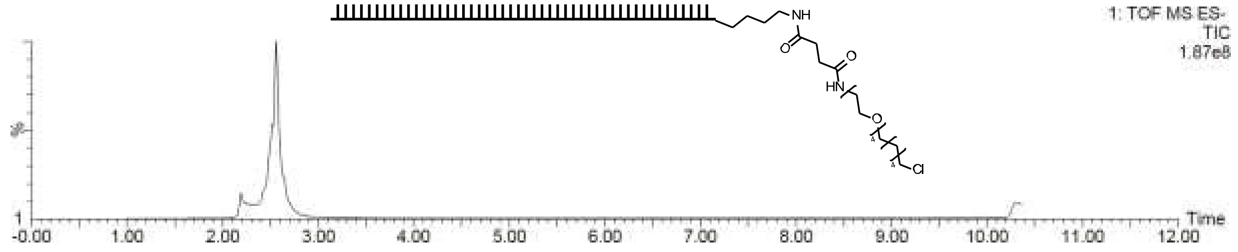

Deconvoluted peak @ 2.6 min. Expected mas0073: 39697, detected: 39698.

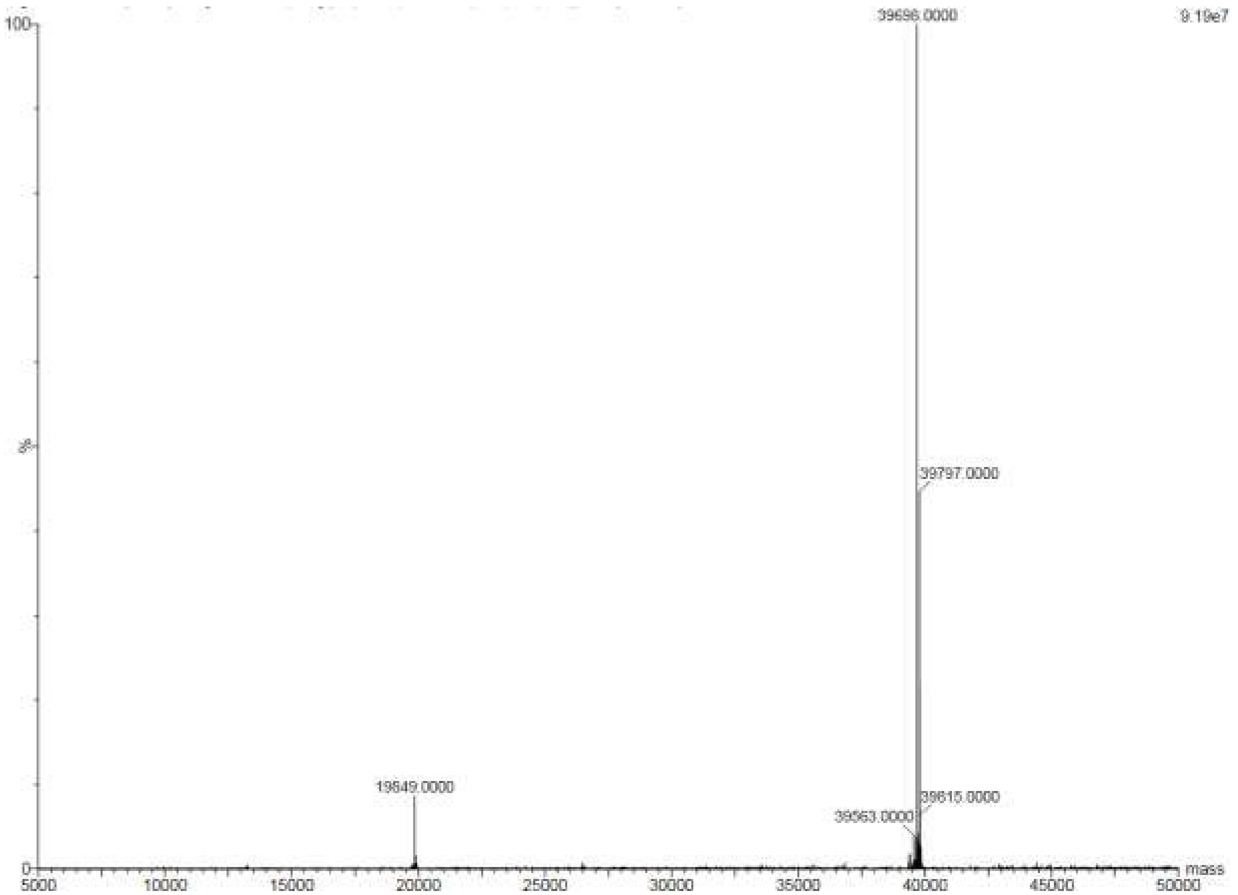

Active Barcode: BC1_TC10-PEG-SH

Deconvoluted Peak. Expected mass: 39621, detected mass: 39621.

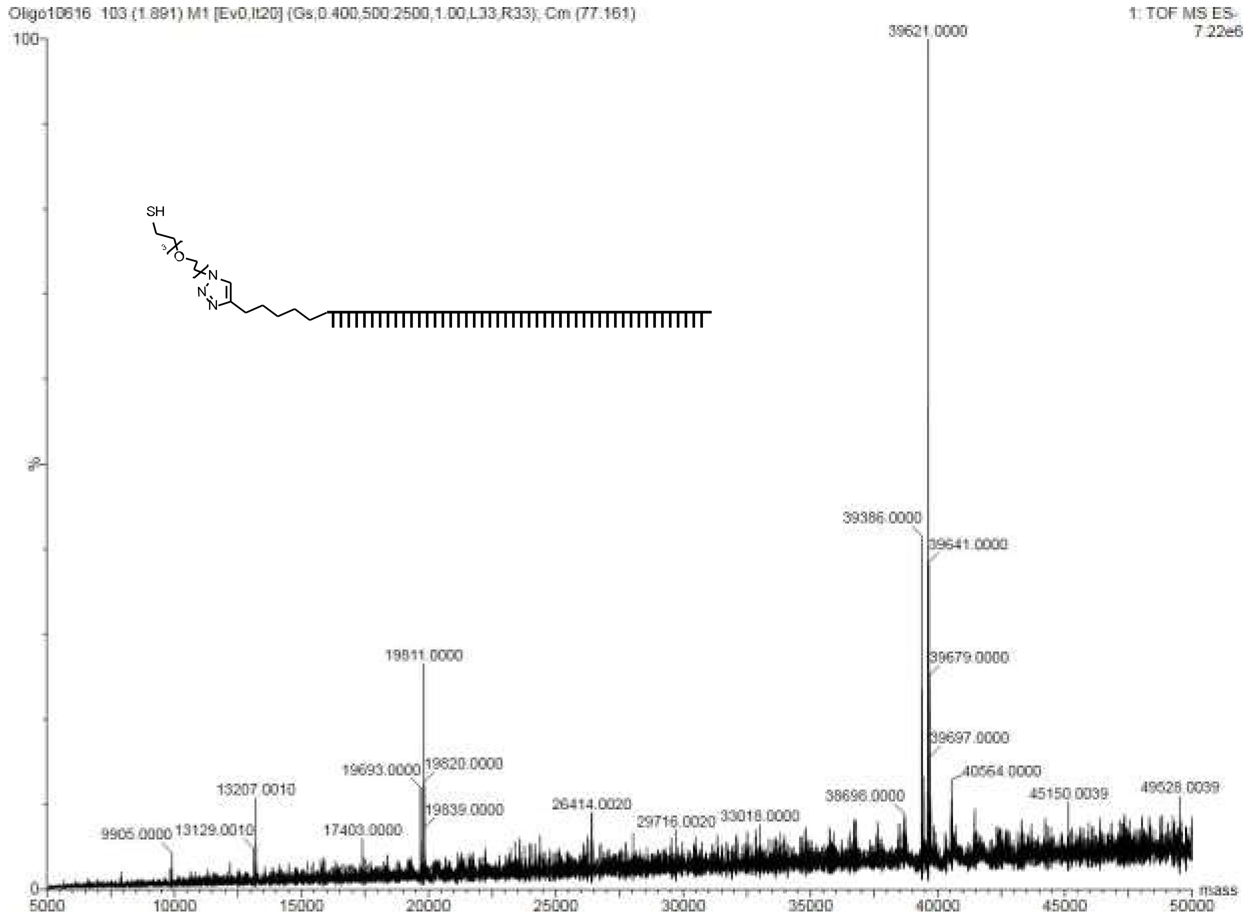

Active Barcode: BC1_TC10-PC-6-NH_2-hexyl-MFP Fwd

TIC Chromatogram

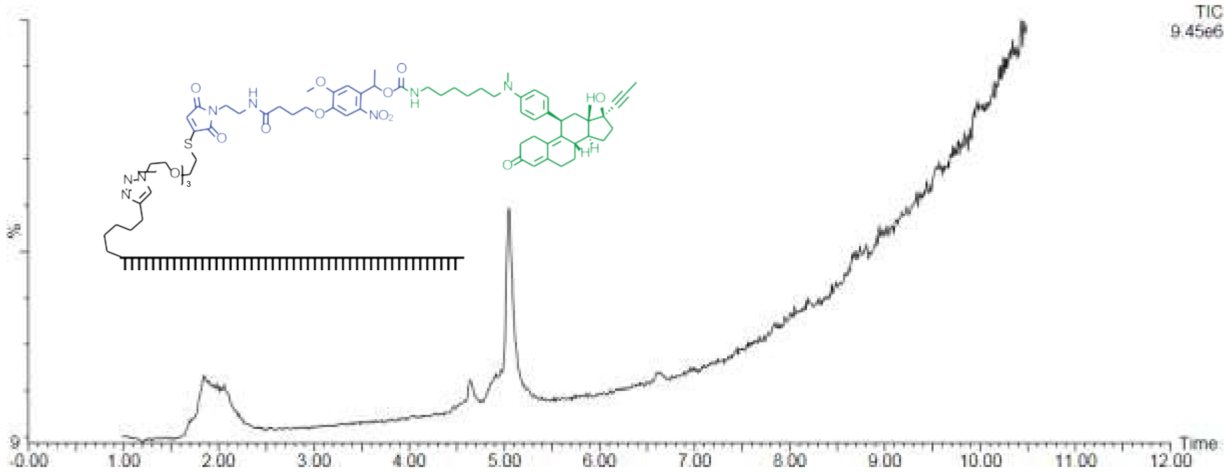

Deconvoluted Peak @ 5 min. Expected mass: 40583, detected mass: 40584

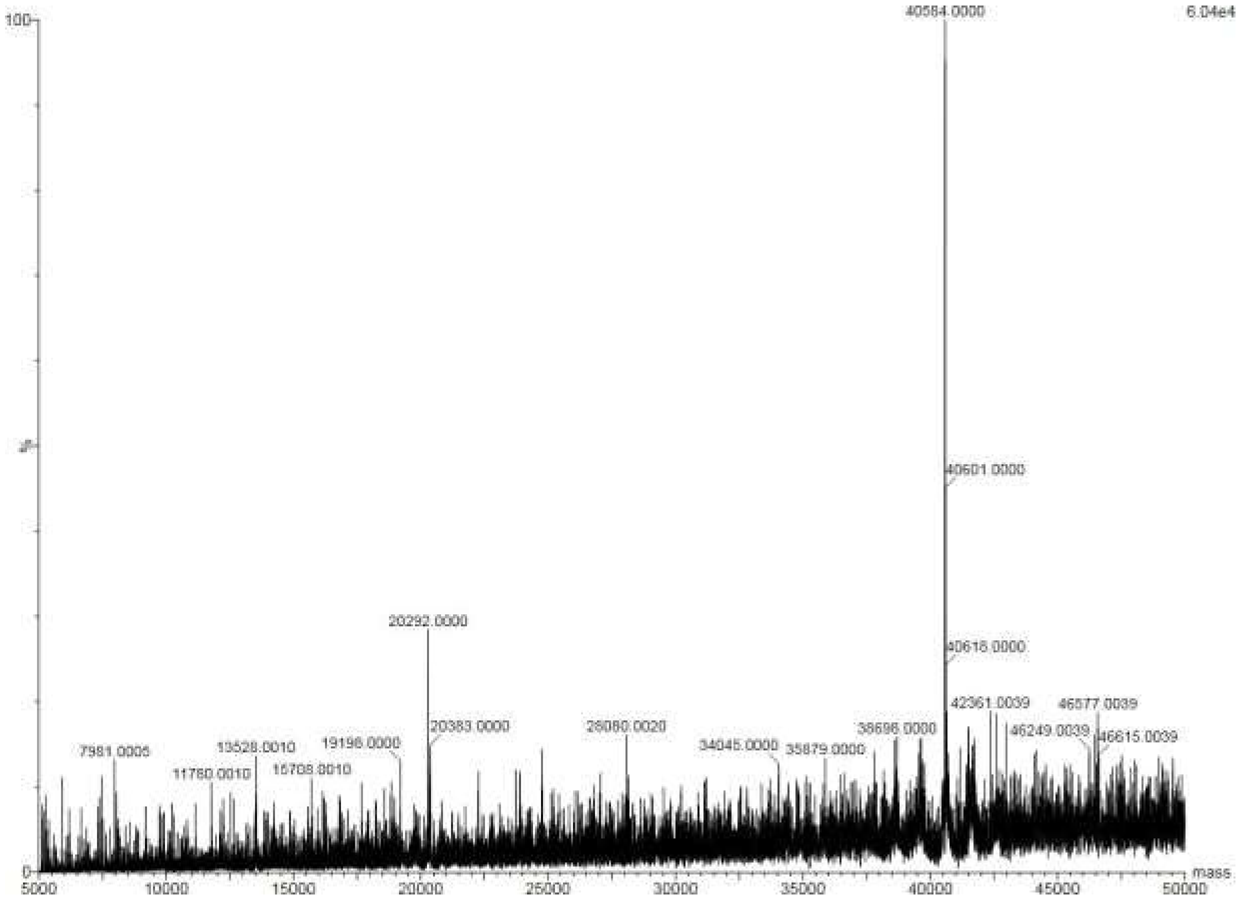

Active Barcode: BC1_TC10-Chloroalkane Rev

Deconvoluted Peak. Expected mass: 39696, detected mass: 39696

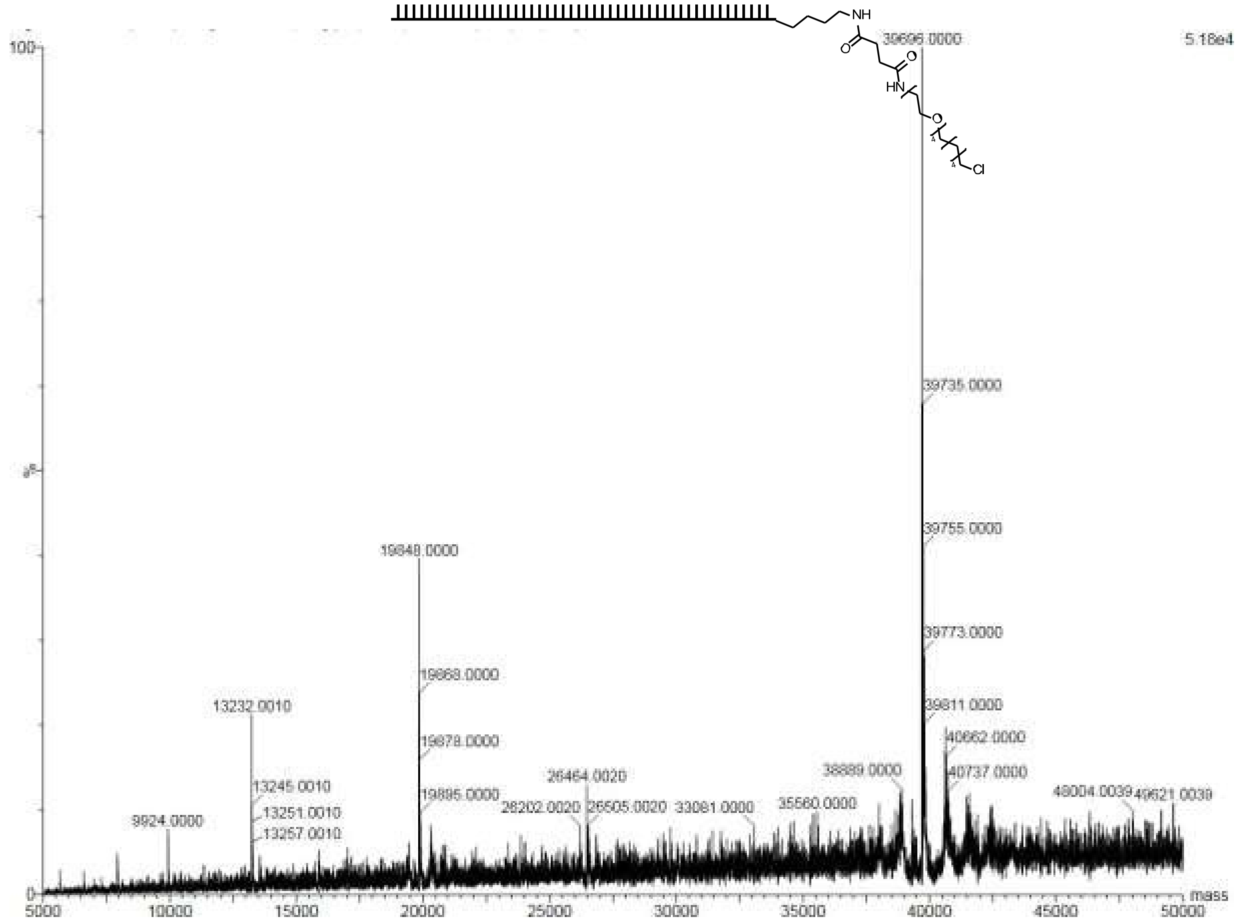

Normalization Barcode: BC2_TC5 Fwd and Rev -chloroalkane

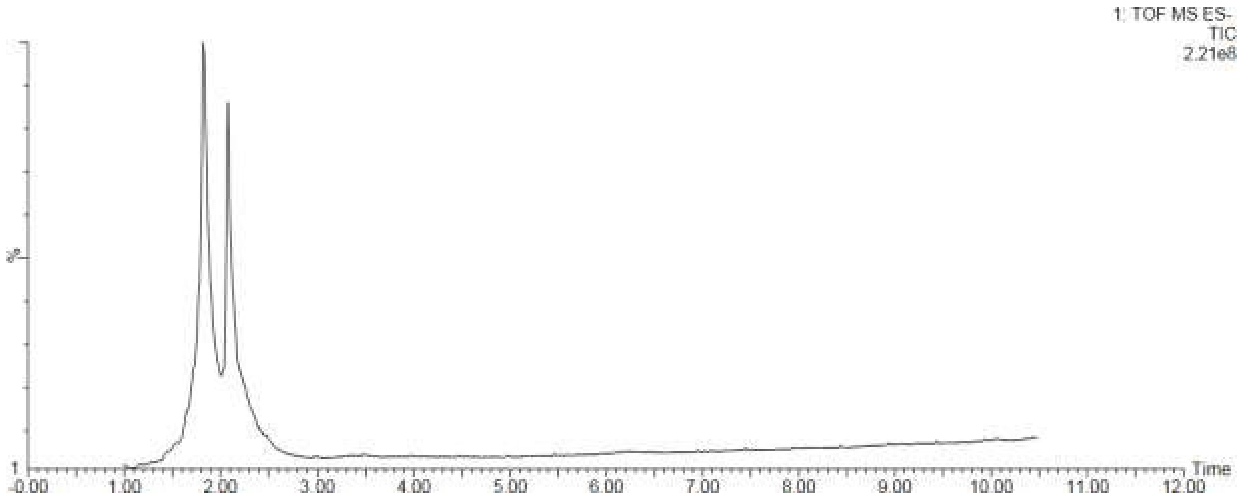

Deconvolution of combined peaks. Expected masses: Fwd: 39218, detected: 39218. Rev-Chloroalkane: 39701, detected:39702.

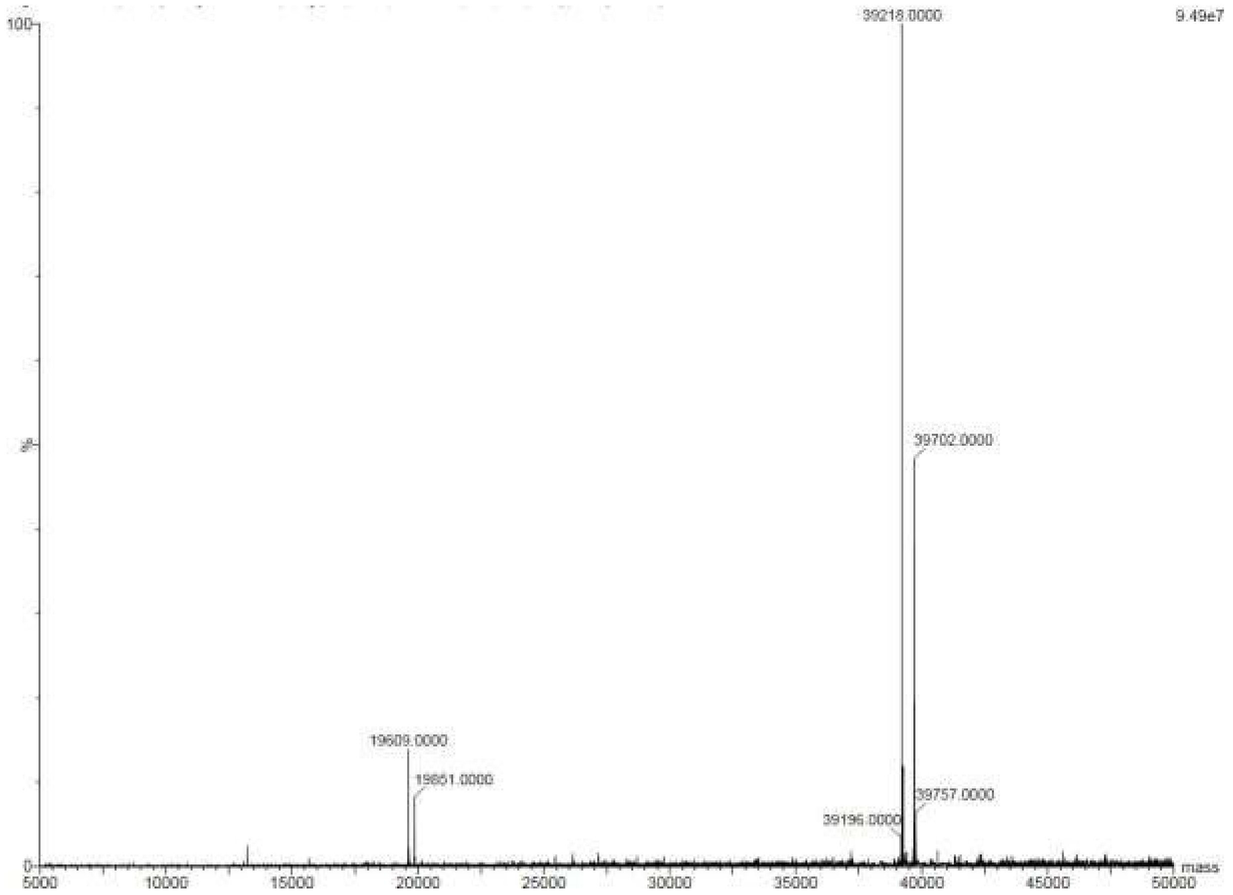

Supplement: CB-006-D4CB00137K-s001 [file CB-006-D4CB00137K-s001.pdf]
